# Synthesis of the furo[2,3-*b*]chromene ring system of hyperaspindols A and B

**DOI:** 10.3762/bjoc.11.29

**Published:** 2015-02-17

**Authors:** Danielle L Paterson, David Barker

**Affiliations:** 1 School of Chemical Sciences, University of Auckland, 23 Symonds St, Auckland, New Zealand

**Keywords:** acylphloroglucinols, bicyclisation, furo[2,3-*b*]chromene, fused ketal

## Abstract

The synthesis of the unique furo[2,3-*b*]chromene ring system found in hyperaspidinols A and B, acylphloroglucinols from *Hypericum chinense* has been achieved in twelve steps. By comparison of the NMR spectra of the synthesized compounds with those of the natural products, a relative stereochemistry is suggested, especially that of the ketal carbon.

## Introduction

**Figure 1:**
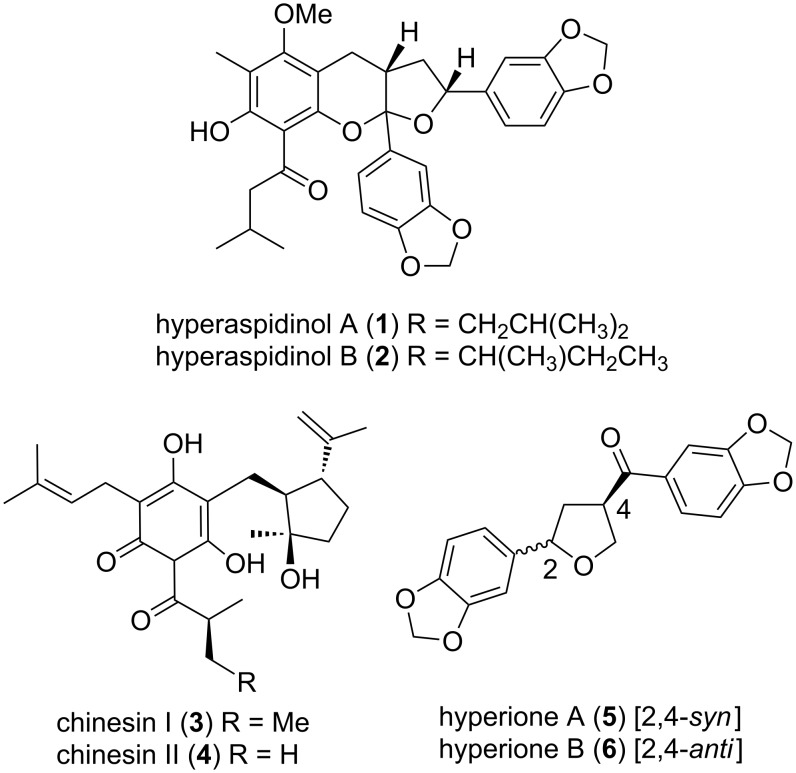
Hyperaspidinols A (**1**) and B (**2**) and other compounds **3-6** from *Hypericum chinense*.

Two novel racemic acylphoroglucinols, hyperaspidinols A (**1**) and B (**2**) (Figure 1) were recently isolated from the leaves of *Hypericum chinense*, a member of the St John’s Wort plants, which contains 490 flowering plants [1]. These plants have been used medicinally for treating illnesses such as hepatitis and depression, and as topical antimicrobials for wounds and snake bites [2–5]. There is great interest in secondary metabolites produced by plants from the *Hypericum* genus due to the bioactivity of many compounds that have been isolated from this source. A wide variety of compounds have been isolated from *H. chinense* including prenylated acylphloroglucinols such as chinesins I (**3**) and II (**4**), xanthones, flavonoids, terpenoids, naphthodianthrones, and norlignans, such as hyperione A (**5**) and B (**6**) [1,6–8]. Chinesins I (**3**) and II (**4**) are acylphloroglucinol derivatives which possess antibacterial and antiviral activities, as well as inhibitory activity on thromboxane A2 and leukotriene D4 [4]. Acylphloroglucinols are known to act as anti-oxidants, by reducing hydroperoxides and hydrogen peroxide, thereby suppressing the formation of the reactive species [9].

**Figure 2:**
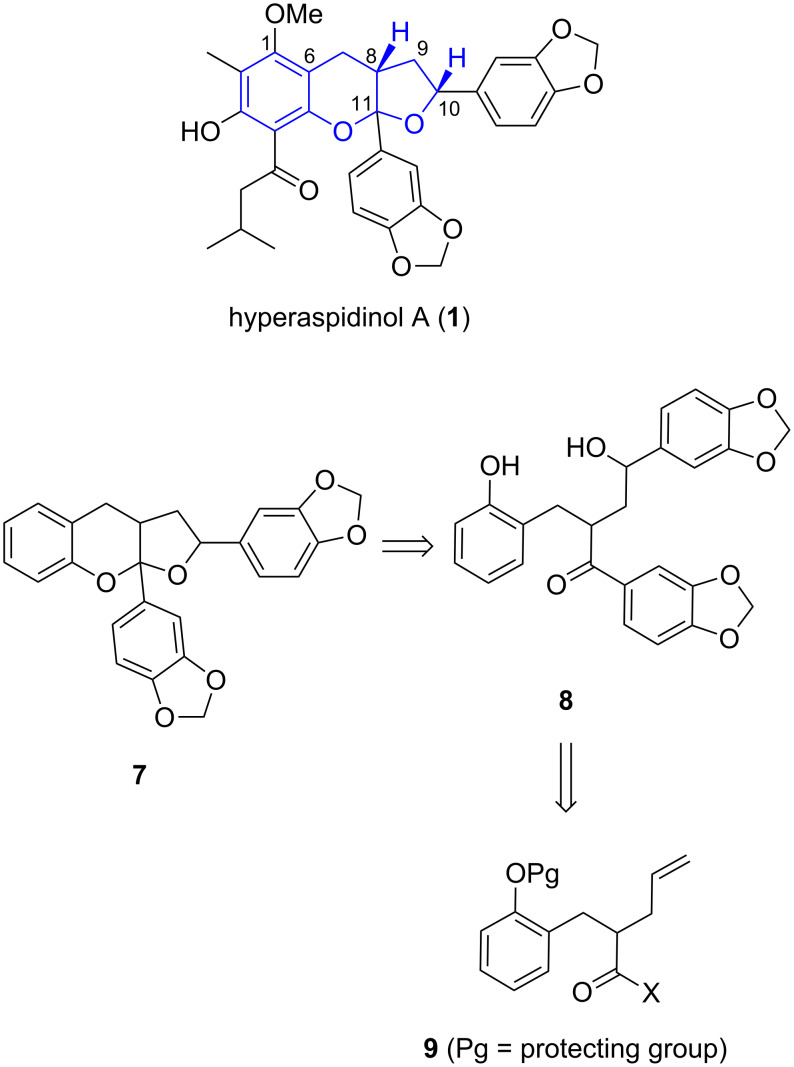
Hyperaspidinol A (**1**), target compound **7** and proposed precursors.

The hyperaspidinols **1** and **2** both possess a highly functionalised furo[2,3-*b*]chromene ring system (Figure 2, highlighted in blue) and differ only with the nature of the ketone side-chain. Furo[2,3-*b*]chromenes have not been reported in any other natural products to date with the closest related system being the chromeno[2,3-*b*]chromenes. Compound which contain this motif, such as albanol A and australisine A, display potent bioactive properties including hypotensive, anticancer, antimicrobial and antimalarial activity [10–12].

The unique ring system found in the hyperaspidinols combined with the potent biological activities of similar structures led us to explore routes to the carboheterocyclic skeleton of these compounds. The relative stereochemistry of the hyperaspidinols was determined using a variety of NMR spectroscopic techniques. Whilst the isolation paper reported a weak ROESY interaction between H-8 and H-10, suggesting a *cis* configuration between these protons, the relationship of the aryl substituents could not be determined (Figure 2) [1]. Our present aim was therefore to prepare furo[2,3-*b*]chromene **7** which contains the unique ring system and aryl subsitutents found in hyperaspidinols **1** and **2** and hopefully use it to determine the complete relative stereochemistry of the natural products.

## Results and Discussion

The proposed route to furo[2,3-*b*]chromene **7** was based around the preparation of benzylic ketone **8** which was hoped to under acidic conditions would undergo cyclisation to give **7** (Figure 2). We have previously reported the formation of a number of diaryl tetrahydrofuran lignans under acidic or buffered conditions utilising the high reactivity of electron-rich benzylic alcohols to assist in the rapid and high yielding formation of the tetrahydrofuran ring [13–18]. The two methylenedioxyphenyl groups in ketone **8** would be added by sequential addition of aryllithiates to both the carbonyl and aldehyde groups, derived from the terminal alkene, of a homoallylic carboxylic acid derivative **9**.

**Scheme 1:**
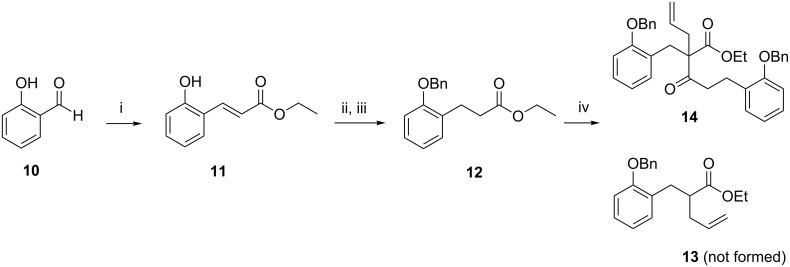
Reagents and conditions: (i) triethylphosphonoacetate, DBU, THF, 48 h, 94%; (ii) H_2_, 10% Pd/C, EtOAc, 3 h, quant; (iii) BnBr, K_2_CO_3_, DMF, 3 h, 88%; (iv) LDA, THF, −78 °C, then allyl bromide, 24 h, 14%.

Our route to ketone **8** began from salicylaldehyde (**10**) which underwent a Horner–Wadsworth–Emmons reaction with triethylphosphonoacetate to give ester **11** [19] in 94% yield (Scheme 1). Hydrogenation of the alkene followed by protection of the phenol gave benzyl ether **12** in 88% yield over two steps. Initially the preparation of alkene **13** through the allylation of ester **12** was attempted, however keto-ester **14** resulting from an additional Claisen condensation was the only isolated product.

**Scheme 2:**
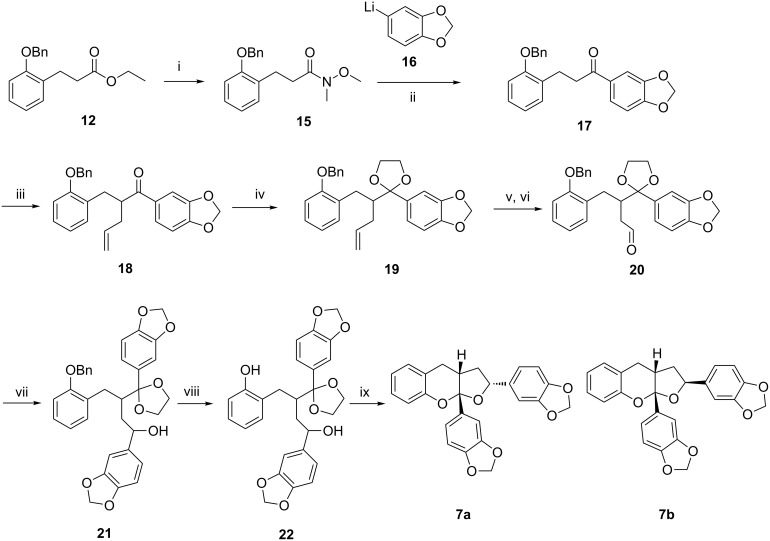
Reagents and conditions: (i) H_3_C(CH_3_O)NH·HCl, *n*-BuLi, THF, −78 °C, 4 h, 81%; (ii) 1-bromo-3,4-methylenedioxybenzene, *t*-BuLi, THF, −78 °C, 3 h, 84%; (iii) NaH, allyl bromide, TBAI, THF, 65 °C, 20 h, 95%; (iv) ethylene glycol, *p*TSA, toluene, reflux, 16 h, 44%; (v) OsO_4_, NMO, *t*-BuOH, H_2_O, THF, 65 h, 80%; (vi) NaIO_4_, MeOH, H_2_O, 3 h, quant; (vii) 1-bromo-3,4-methylenedioxybenzene, *t*-BuLi, THF, −78 °C, 19 h, 87% 1:1 diastereoisomers; (viii) H_2_, 10% Pd/C, MeOH, 4 h, quant; (ix) 1:1 2 M HCl (aq):THF, 22 h, **7a** 52%, **7b** 32%.

It was therefore decided to functionalise the ester moiety first. This was achieved by direct conversion of ester **12** into Weinreb amide **15** in 81% yield, followed by addition of lithiate **16** (formed from 1-bromo-3,4-methylenedioxybenzene and *t*-butyllithium) to give ketone **17** in 84% yield (Scheme 2). Allylation of ketone **17** proved problematic with the use of strong bases such as LDA and LiHMDS, giving none of the desired product. However, the use of allyl bromide and NaH in the presence of TBAI, in THF at reflux, gave the desired alkene **18** in 95% yield. Protection of the ketone in **18** as the cyclic ketal **19** was achieved in 44% yield using excess ethylene glycol and *p*TSA and despite attempting a variety of alternate conditions [20–23] this yield could not be improved without degradation of both the starting material **18** and product **19**. Dihydroxylation of **19**, followed by oxidative cleavage of the resultant diol gave aldehyde **20** in 80% yield over two steps. Addition of lithiate **16** to aldehyde **20** gave alcohol **21** in 87% yield as an inseparable 1:1 mixture of diastereoisomers. Hydrogenolysis of the benzyl ether in **21** gave ketal-diol **22** in quantitative yield. Finally, stirring of ketal **22** in a 1:1 2 M HCl (aq):THF resulted in removal of the ketal protecting group, giving ketone **8**, which under the acidic condition immediately cyclized giving furo[2,3-*b*]chromenes **7a** and **7b** in a 1.6:1 ratio, in an overall 84% yield. Separation of the diastereoisomers **7a** and **7b** was achieved using column chromatography and allowed characterisation of the individual isomers.

Comparison of the ^1^H and ^13^C NMR data of isomers **7a** and **7b** showed strong similarities between **7a** and the reported data [1] for hyperaspidinols A (**1**) and B (**2**) whilst isomer **7b** showed clear differences in both the chemical shift and multiplicities in the furo[2,3-*b*]chromene rings (see Supporting Information File 1 for a complete table of NMR data).

**Figure 3:**
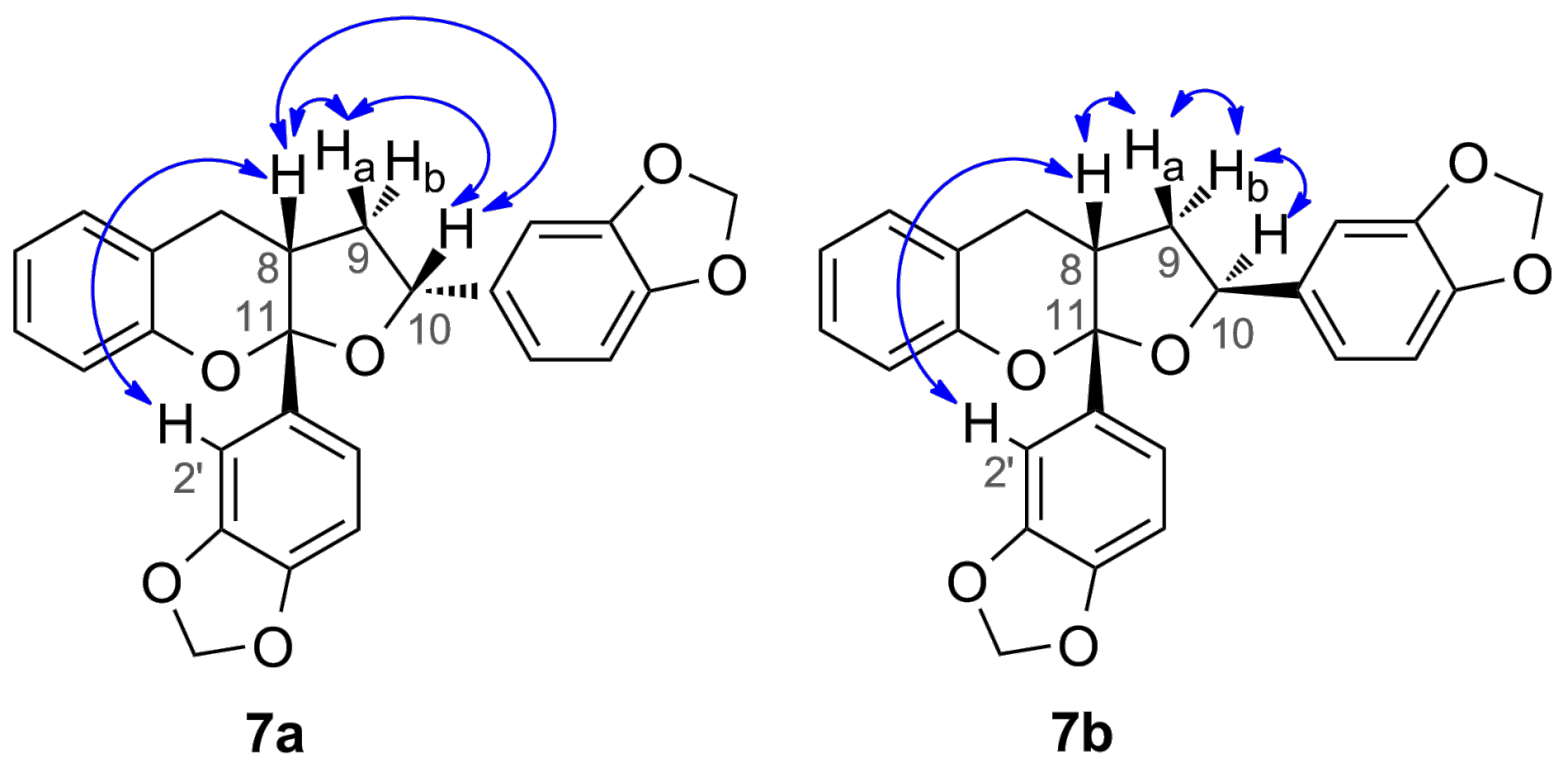
NOESY correlations in isomers **7a** and **7b**.

Extensive use of 2D NMR techniques, in particular a NOESY, allowed the complete relative stereochemistry of both **7a** and **7b** to be determined (Figure 3). In isomer **7a** NOESY correlations between H-8 and H-10 showed the *syn* relationship between these two protons. An additional NOESY correlation between H-8 and H-2′ showed the *syn* relationship between these two groups and thus the *trans* relationship between the two methylenedioxyphenyl groups at C-10 and C-11.

**Figure 4:**
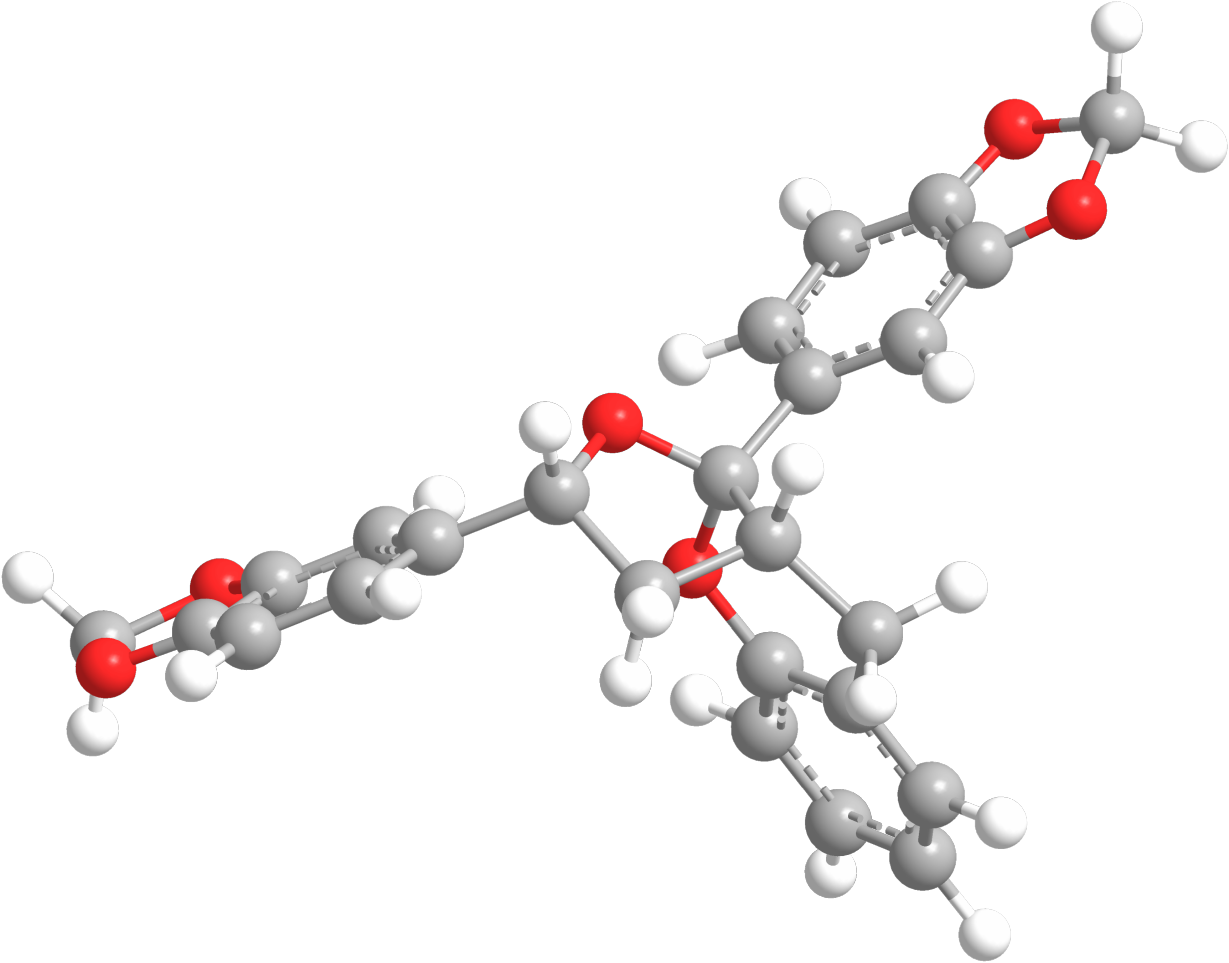
3D representation of **7a**.

**Figure 5:**
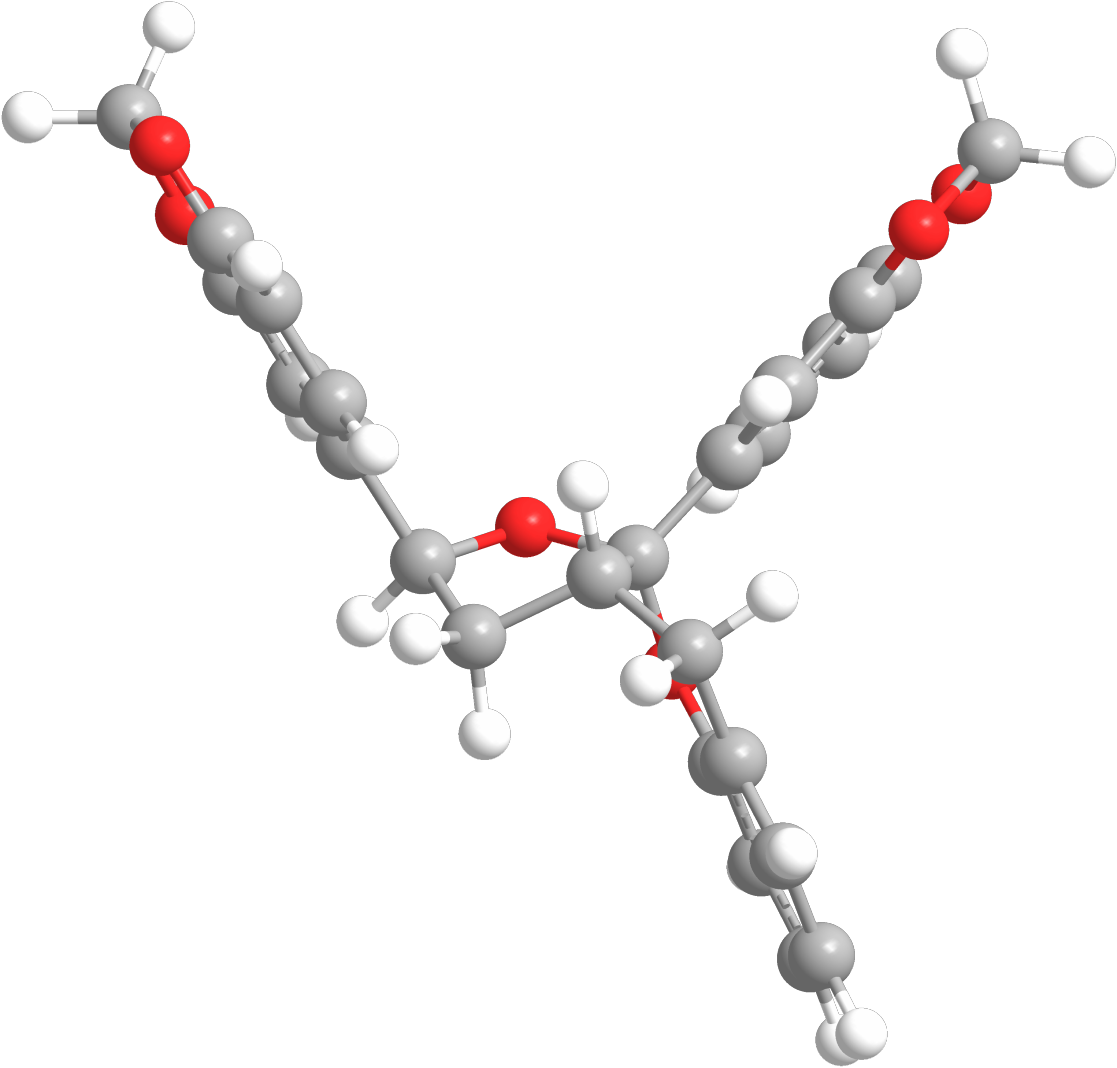
3D representation of **7b**.

The NOESY spectrum of **7b** exhibited a NOESY correlation between H-8 and H-2′ showed the *syn* relationship between these two groups but no correlation between H-8 and H-10 was observed, suggesting **7b** to be the C-10 epimer of **7a**. A correlation of H-10 to the H-9b, which was on the opposite face to H-8 further suggested this. Further analysis of the conformations of **7a** and **7b** was achieved by examination of the coupling constants in the furo[2,3-*b*]chromene ring. The use of coupling constants to determine stereochemistry in five-membered rings is often extremely difficult, due to the large number of conformations available [24–26]. However, with the relatively fixed geometry for the five membered ring in **7a** and **7b** due to the fused chroman ring and the quarternary centre at C-11, analysis of the coupling constants was considered viable. In isomer **7a** the coupling constant between H-9a and both H-8 and H-10 was 6.5 Hz, which corresponds to a ca. 50° dihedral angle between the protons, placing all three on the same face of the five membered ring (Figure 4). Additionally H-9b had coupling constants of 10.5 Hz and 12.6 Hz between H-8 and H-10, respectively, showing an *anti*-relationship between these protons [25]. In isomer **7b** the coupling constant between H-8 and H-9b (9.5 Hz) represent a ca. 170° dihedral angle between these protons whilst the coupling constant between H-9b and H-10 (4.5 Hz) represent a ca. 40° dihedral angle or *syn* relationship between these two protons (Figure 5) [26]. Overall this represents an *anti-*relationship between H-8 and H-10 confirming **7b** to be the C-10 epimer of **7a**.

**Figure 6:**
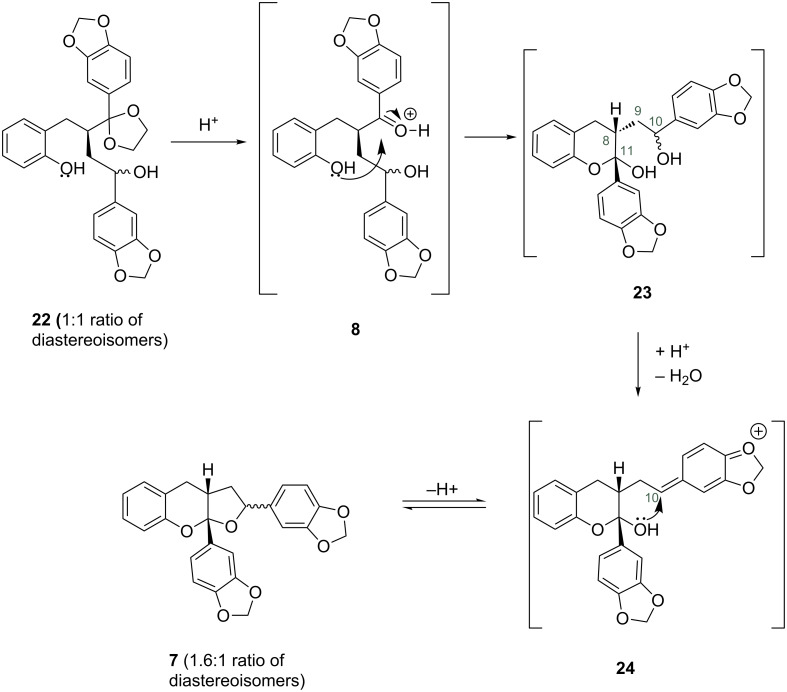
Possible mechanism for the formation of furo[2,3-*b*]chromenes **7a** and **7b**.

The ratio of diastereoisomers in ketal **22** was 1:1, however in the cyclised products **7** the ratio of diastereoisomers **7a** to **7b** was 1.6:1. This suggests partial isomerisation of the stereochemistry at C-10 during the formation of the furo[2,3-*b*]chromene. Indeed, one possible mechanism for the formation of isomers **7a** and **7b** is initial protonation of ketone **8**, attack of phenol on the activated carbonyl resulting in formation of chroman-hemiketal **23**, where the large C-11 aryl group and arylethyl substituent at C-8 adopt an *anti* relationship (Figure 6). Loss of stereochemistry at C-10 is most likely due to the formation of a quinone methide, such as **24**, which upon attack by the C-11 alcohol gives a mixture of isomers with the isomer **7a** being favoured over the more sterically congested **7b**. We have noticed a similar process in the synthesis of other THF lignans [15–16]. To test this hypothesis we stirred a sample of isomer **7b** in 1:1 2 M HCl (aq)/THF and found that after 24 hours obtained a mixture of **7a** to **7b** with isomer **7a** being predominant. This highlights the reversibility of the ring closure and preferred formation of isomer **7a**.

## Conclusion

In conclusion, the first synthesis of the furo[2,3-*b*]chromene ring system found in hyperaspidinols A (**1**) and B (**2**) has been achieved. Analysis of the NMR of synthetic furo[2,3-*b*]chromenes **7a** and **7b** and comparison to the data of **1** and **2** provides clues to the relative stereochemistry of all substituents on the natural products. The route developed allows easy introduction of alternate aryl substituents at the C-10 and C-11 positions, whilst replacing salicylaldehyde **10** with a more functionalised aldehyde could allow for the preparation of a number of highly functionalised furo[2,3-*b*]chromenes, including the natural products **1** and **2**.

## Supporting Information

File 1 Experimental procedures, characterisation data of new compounds and NMR tables of **7a** and **7b**.

File 2 ^1^H/^13^C NMR spectra.
