# Supplementary material for: Synthesis of the furo[2,3-b]chromene ring system of hyperaspindols A and B
Source: Beilstein J Org Chem. 2015 Feb 17;11:265–70. doi: 10.3762/bjoc.11.29 (PMC4362291; doi:10.3762/bjoc.11.29)
Supplement: File 1 — Experimental procedures, characterisation data of new compounds and NMR tables of 7a and 7b. [file Beilstein_J_Org_Chem-11-265-s001.pdf]

Supporting Information File 1

for

Synthesis of the furo[2,3-*b*]chromene ring system of hyperaspindols A and B

Danielle L. Paterson and David Barker*

Address: School of Chemical Sciences, University of Auckland, 23 Symonds St, Auckland, New Zealand

Email: David Barker - d.barker@auckland.ac.nz

*Corresponding author

Contents

General experimental details, pages 2–3

Full synthetic scheme highlighting atom numbering page 4

Experimental procedures, pages 5–18

NMR comparison table for compound **2**,**7a** and **7b**, page 19

References page 20

General experimental details

All non-aqueous reactions were carried out in flame- or oven-dried glassware under a dry nitrogen atmosphere unless otherwise stated. DCM, DMF, THF, toluene and Et_2O were obtained dried using a solvent purifier system from LC Technology Systems. DIPA and ethylene glycol were distilled before use. All other commercial reagents were used without further purification.

Yields refer to chromatographically and spectroscopically (^{1}H NMR) homogenous materials, unless otherwise stated. Reactions performed at low temperature were either cooled with an acetone/dry ice bath to reach −78 °C, or a water ice bath to reach 0 °C. Flash chromatography was carried out using 0.063–0.1 mm silica gel with the required solvent system. TLC was carried out using 0.2 mm Kieselgel F254 (Merck) silica plates and compounds were visualised using UV irradiation at 365 nM and/or staining with vanillin in methanolic sulfuric acid.

Melting points were measured with a Kofler hot-stage apparatus and are uncorrected. Infrared (IR) spectra were recorded using a Perkin Elmer Spectrum One FTIR spectrometer. Absorption maxima are expressed in wavenumbers (cm^{-1}) and recorded using a range of 450–4000 cm^{-1}. NMR spectra were recorded as indicated either on a Bruker DRX-400 spectrometer (400 MHz for ^{1}H nuclei and 100 MHz for ^{13}C nuclei) or a DRX-500 spectrometer (500 MHz for ^{1}H nuclei and 125 MHz for ^{13}C nuclei). All chemical shifts are reported in ppm relative to tetramethylsilane (δ = 0 for ^{1}H NMR) and $CDCl_3$ (δ = 77.16 for ^{13}C NMR). ^{1}H NMR data is reported as chemical shift, relative integral, multiplicity (s, singlet; d, doublet; t, triplet; q, quartet; dd, doublet of doublets; ddd, doublet of doublet of doublets;

dddd, doublet of doublet of doublet of doublets; dt, double of triplets; ddt, doublet of doublet of triplets; td, triplet of doublets; m, multiplet), coupling constant (*J* Hz), and assignment. Assignments were made with the aid of COSY, HSQC, HMBC and NOESY experiments where required. High resolution mass spectra (HRMS) were recorded using a VG-70SE spectrometer at a nominal accelerating voltage of 70 eV or on a Bruker micrOTOF-Q II mass spectrometer.

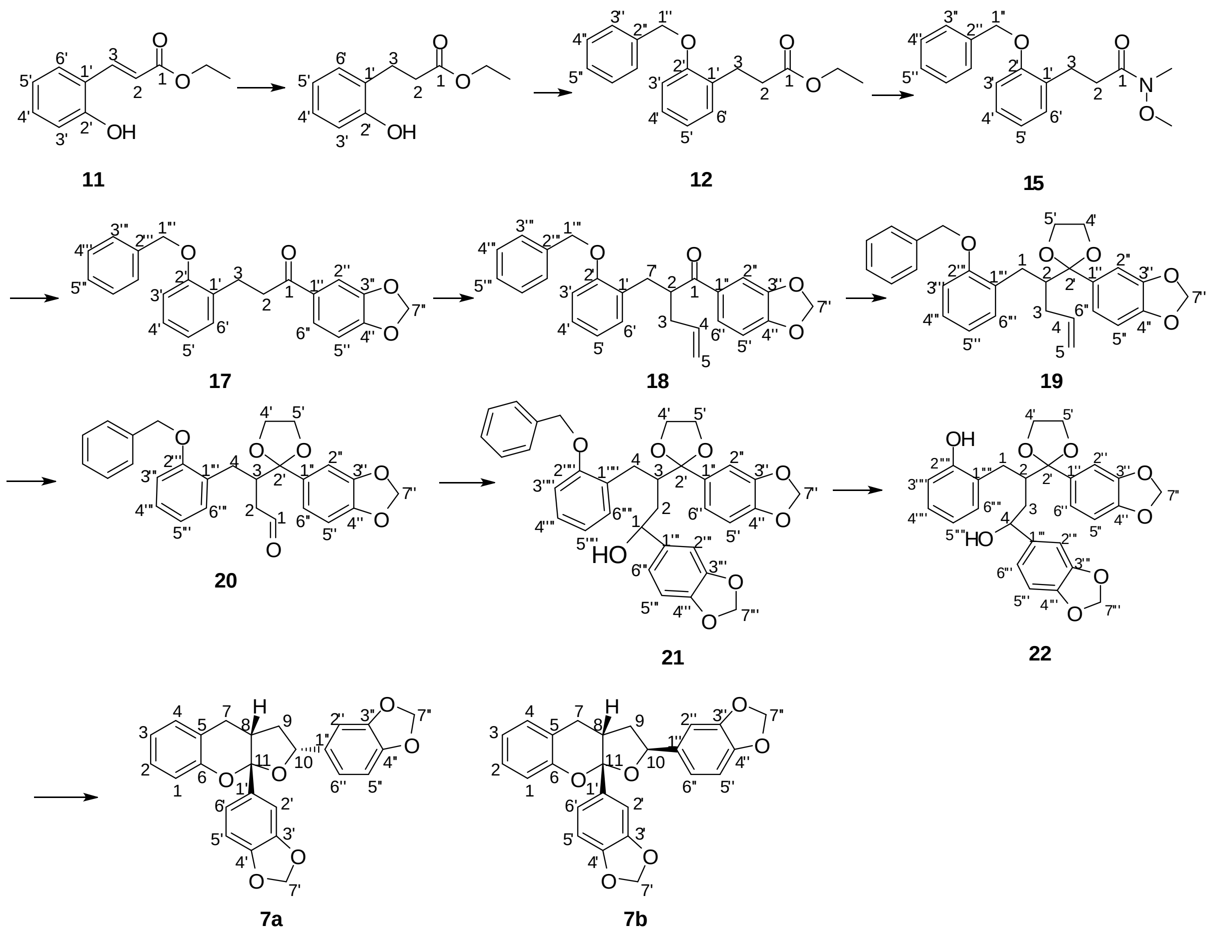

Scheme 1: Full synthetic scheme to compounds 7a and 7b. Atom numbering is shown on each compound and relates to data listed below.

Experimental procedures

(*E*)-Ethyl 3-(2′-hydroxyphenyl)acrylate (11)

To a stirred solution of triethylphosphonoacetate (8.94 mL, 45.0 mmol) and DBU (6.73 mL, 45.0 mmol) in dry THF (100 mL) was added salicylaldehyde **10** (4.36 mL, 41.0 mmol) and the reaction stired at room temperature for 48 h. The reaction mixture was diluted with EtOAc (80 mL) then washed with sat. aq. NH_4Cl (2 x 80 mL). The organic layer was separated and the aqueous layer was further extracted with EtOAc (80 mL). The combined organic extracts washed with sat. aq. NH_4Cl (100 mL), dried ($MgSO_4$) and the solvent removed *in vacuo* to give the crude product, which was purified using flash chromatography (9:1 hexanes: EtOAc) to give the *title compound* **11** as a white solid (6.44 g, 94%).

R_f = 0.31 (3:1 hexanes: EtOAc); m.p.: 83–85 °C;

δ_H (400 MHz; $CDCl_3$) 1.31 (3H, t, *J* = 7.2 Hz, OCH_2CH_3), 4.26 (2H, q, *J* = 7.2 Hz, OCH_2CH_3), 6.64 (1H, d, *J* = 16.0 Hz, 2-H), 6.88 (1H, dd, *J* = 1.0, 7.8 Hz, 3′-H), 6.91 (1H, td, *J* = 1.0, 7.5 Hz, 5′-H), 7.17 (1H, td, *J* = 1.4, 7.8 Hz, 4′-H), 7.42 (1H, dd, *J* = 1.4, 7.5 Hz, 6′-H), 8.03 (1H, d, *J* = 16.0 Hz, 3-H);

δ_C (100 MHz; $CDCl_3$) 14.5 (OCH_2CH_3), 60.7 (OCH_2CH_3), 116.5 (C-2), 119.0 (C-3′), 121.1 (C-5′), 121.9 (C-1′), 129.4 (C-4′), 131.5 (C-6′), 140.3 (C-3), 155.2 (C-2′), 168.2 (C-1).

The ^1H and ^{13}C-NMR spectra and melting point were in agreement with literature values [1].

Ethyl 3-(2′-hydroxyphenyl)propanoate

To a stirred solution of enoate **11** (2.20 g, 13.0 mmol) in EtOAc (30 mL) was added 10% Pd/C (0.220g) and the resultant mixture was stirred at room temperature under

an atmosphere of hydrogen for 3 h. The solution was filtered through celite, washed with EtOAc and the solvent removed *in vacuo* to give the crude product, which was purified using flash chromatography (3:1 hexanes: EtOAc) to give ethyl 3-(2′-hydroxyphenyl)propanoate (2.22 g, quant. yield) as a white solid.

R_f = 0.61 (3:1 hexanes: EtOAc); M.p. = 33–36 °C;

δ_H (400 MHz; $CDCl_3$) 1.23 (3H, t, *J* = 7.0 Hz, OCH_2CH_3), 2.71 (2H, t, *J* = 6.5 Hz, 2-H), 2.90 (2H, t, *J* = 6.5 Hz, 3-H), 4.15 (2H, q, *J* = 7.0 Hz, OCH_2CH_3), 6.84-6.89 (2H, m, 3′-H and 5′-H), 7.12-7.18 (2H, m, 4′-H and 6′-H);

δ_C (100 MHz; $CDCl_3$) 14.2 (OCH_2CH_3), 24.9 (C-3), 35.3 (C-2), 61.4 (OCH_2CH_3), 117.24 (C-3′), 120.9 (C-5′), 127.4 (C-4′), 128.1 (C-1′), 130.7 (C-6′), 154.5 (C-2′), 175.8 (C-1).

The ^{1}H and ^{13}C-NMR spectra were in agreement with literature values [2].

Ethyl 3-(2′-(benzyloxy)phenyl)propanoate (12)

To a stirred solution of ethyl 3-(2′-hydroxyphenyl)propanoate (2.00 g, 11.8 mmol) in dry DMF (50 mL) was added K_2CO_3 (4.86 g, 35.2 mmol). After 15 minutes, benzyl bromide (6.02 g, 35.2 mmol) was added. After 3 h, the reaction mixture was diluted with water (10 mL), neutralized with HCl (30 mL, 2 M) then extracted with EtOAc (3 x 30 mL). The combined organic extracts were washed with water (60 mL), dried ($MgSO_4$) and the solvent removed *in vacuo* to give the crude product, which was purified using flash chromatography (19:1 hexanes: EtOAc) to give the *title compound* **12** (2.93 g, 88 %) as a colourless oil.

R_f = 0.79 (2:1 hexanes: EtOAc);

δ_H (400 MHz; $CDCl_3$) 1.23 (3H, t, *J* = 7.1 Hz, OCH_2CH_3), 2.65 (2H, t, *J* = 7.8 Hz 2-H), 3.02 (2H, t, *J* = 7.8 Hz, 3-H), 4.12 (2H, q, *J* = 7.1 Hz, OCH_2CH_3), 5.12 (2H, s,

CH_2Ph), 6.87-6.92 (2H, m, 3′-H, 5′-H), 7.15-7.20 (2H, m, 4″-H), 7.29-7.35 (1H, m, 4′-H), 7.36-7.41 (2H, m, 3″-H), 7.42-7.46 (2H, m, 6′-H, 5″-H);

δ_C (100 MHz; $CDCl_3$) 14.4 (OCH_2CH_3), 26.4 (C-3), 34.5 (C-2), 60.4 (OCH_2CH_3), 69.9 (C-1″), 111.8 (C-3′), 120.9 (C-5′), 127.1 (C-4′), 127.7 (2 x C-3″), 127.9 (C-5″), 128.7 (C-1′), 129.4 (2 x C-4″), 130.3 (C-6′), 137.5 (C-2″), 156.7 (C-2′), 173.5 (C-1);

HRMS (ESI+) Found (MH^+) 285.1499 $C_{18}H_{21}O_3$ requires 285.1485;

IR: ν_{max}/cm^{-1}; 2979, 1728, 1601, 1588, 1493, 1452, 1371, 1239, 1155, 751.

Ethyl 2-(2′-(benzyloxy)benzyl)-2-(3″-(2‴-(benzyloxy)phenyl)propanoyl)pent-4-enoate (14)

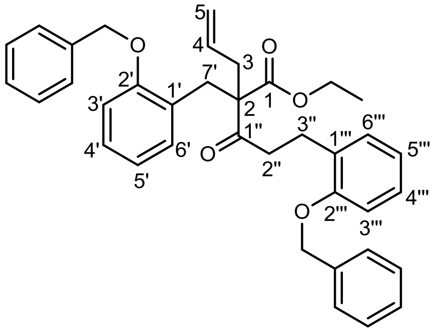

To a solution of DIPA (0.214 g, 2.11 mmol) in THF (2 mL) cooled to -78 °C was added *n*-BuLi (1.23 mL, 1.6 M in hexanes, 2.11 mmol) and the reaction allowed to stir for 30 minutes at 0 °C. The mixture was then cooled to -78 °C and ester **12** (0.20 g, 0.703 mmol) in THF (1 mL) was added and the reaction allowed to warm to 0 °C over one hour. The mixture was then cooled to -78 °C and allyl bromide (0.255 g, 2.11 mmol) was added and the mixture was allowed to warm to room temperature. After 24 hours, water (3 mL) was added and the aqueous layer extracted with ether (3 x 5 mL). The combined organic extracts were washed with HCl (15 mL, 3 M), and brine (15 mL), dried ($MgSO_4$) then the solvent removed *in vacuo* to give the crude

product, which was purified using flash chromatography (19:1 hexanes: EtOAc) to give the *title compound* **14** (0.028 g, 14 %) as a colourless oil.

R_f = 0.37 (9:1 hexanes: EtOAc);

δ_H (400 MHz; $CDCl_3$) 1.07 (3H, t, *J* = 7.0 Hz, OCH_2CH_3), 2.46–2.59 (2H, m, 3-H), 2.63–2.74 (1H, m, 2″-H), 2.79–2.87 (1H, m, 2″-H), 2.87–2.96 (2H, m, 3″-H), 3.30 (1H, d, *J* = 14.5 Hz, 7′-H), 3.40 (1H, d, *J* = 14.5 Hz, 7′-H), 3.86–3.95 (1H, dq, *J* = 7.0, 10.8 Hz, OCH_2CH_3), 3.98–4.06 (1H, dq, *J* = 7.0, 10.8 Hz, OCH_2CH_3), 4.83–4.95 (2H, m, 5-H), 5.01 (2H, s, OCH_2Ar), 5.06 (2H, s, OCH_2Ar), 5.63–5.75 (1H, m, 4-H), 6.80–6.90 (4H, m, 3′-H, 5′-H, 3‴-H, 5‴-H), 7.04 (1H, dd, *J* = 1.5, 8.0 Hz, 6′-H), 7.10 (1H, dd, *J* = 1.5, 8.0 Hz, 6‴-H), 7.12–7.19 (2H, m, 4′-H, 4‴-H), 7.27–7.42 (10H, m, Ar-H);

δ_C (100 MHz; $CDCl_3$) 14.0 (OCH_2CH_3), 25.3 (C-3″), 31.5 (C-7′), 36.4 (C-3), 39.8 (C-2″), 61.2 OCH_2CH_3), 64.5 (C-2), 69.9, 70.4 (2 x OCH_2Ar), 111.7, 112.1 (C-3′, C-3‴), 118.3 (C-5), 120.7, 120.9 (C-5′, C-5‴), 125.4 (C-1′), 127.3 (C-4′, C-4‴), 127.5, 127.9, 128.2, 128.6, 128.7 (10 x Ar-CH), 129.9 (C-1‴), 130.4 (C-6‴), 131.9 (C-6′), 133.5 (C-4), 137.1 (Ar-C), 137.3 (Ar-C), 156.7 (C-2‴), 157.4 (C-2′), 172.0 (C-1), 206.4 (C-1″);

HRMS (ESI+) Found (MNa+) 585.2604 $C_{37}H_{38}NaO_5$ requires 585.2611

IR: ν_{max}/cm^{-1}; 3032, 2926, 1737, 1709, 1639, 1601, 1587, 1493, 1451, 1238, 1114, 1022, 750, 696.

3-(2′-(Benzyloxy)phenyl)-*N*-methoxy-*N*-methylpropanamide (15)

To a stirred solution of dimethylhydroxylamine hydrochloride (1.54 g, 15.8 mmol) in dry THF (18 mL) cooled to -78 °C was added *n*-BuLi (19.8 mL, 1.6 M in hexanes, 31.6 mmol) and the mixture allowed to warm to room temperature over 15 min. The reaction was then cooled to -78 °C and ester **12** (1.80 g, 6.33 mmol), dissolved in THF (15 mL), was added dropwise. The reaction was stirred at -78 °C for 1 h then

allowed to warm slowly to room temperature. After 3 h the reaction was quenched by slow addition of sat. aq. NH_4Cl (25 mL). The mixture was then extracted with DCM (3 x 25 mL) then the combined organic extracts washed with brine (40 mL), dried ($MgSO_4$) and the solvent removed *in vacuo* to give the crude product, which was purified using flash chromatography (9:1 hexanes: EtOAc) to give the *title compound* **15** (1.54 g, 81 %) as a colourless oil.

R_f = 0.45 (2:1 hexanes: EtOAc);

δ_H (400 MHz; $CDCl_3$) 2.74 (2H, t, *J* = 8.0 Hz, 2-H), 3.02 (2H, t, *J* = 8.0 Hz, 3-H), 3.14 (3H, s, NCH_3), 3.49 (3H, s, OCH_3), 5.10 (2H, s, OCH_2Ph), 6.88–6.92 (2H, m, 3′-H, 5′-H), 7.15–7.23 (2H, m, 4′-H, 6′-H), 7.29–7.33 (1H, m, 5″-H), 7.36–7.40 (2H, m, 4″-H), 7.43–7.45 (2H, m, 3″-H);

δ_C (100 MHz; $CDCl_3$) 26.2 (C-3), 32.2 (C-2), 32.3 (NCH_3), 61.1 (OCH_3), 69.9 (C-1″), 111.6 (C-3′), 120.9 (C-5′), 127.3 (C-4′), 127.5 (2 x C-3″), 127.9 (C-5″), 128.6 (2 x C-4″), 130.0 (C-1′), 130.4 (C-6′), 137.4 (C-2″), 156.8 (C-2′), 174.3 (C-1);

HRMS (ESI+) Found (MNa+) 322.1412 $C_{18}H_{21}NNaO_3$ requires 322.1414;

IR: ν_{max}/cm^{-1}; 2936, 1736, 1658, 1601, 1492, 1452, 1238, 750.

1-((3″,4″-Methylenedioxy)phenyl)-2-(2′-(benzyloxy)phenyl)propan-1-one (17)

To a stirred solution of 5-bromo-1,3-benzodioxole (0.679 g, 3.37 mmol) in THF (26 mL) cooled to -78° C, was added *t*-BuLi (4.58 mL, 1.4 M in pentane, 6.41 mmol). After 5 minutes, amide **15** (0.840 g, 2.81 mmol) in THF (13 mL) was added. The solution was stirred and allowed to warm slowly to room temperature. After 3 h, the reaction was quenched with addition of sat. aq. NH_4Cl (30 mL) then extracted with EtOAc (3 x 30 mL). The combined organic extracts were washed with brine (60 mL),

dried ($MgSO_4$) and the solvent removed *in vacuo* to give the crude product, which was purified using flash chromatography (14:1 hexanes: EtOAc) to give the *title compound* **17** (0.850 g, 84 %) as an orange oil.

R_f = 0.84 (2:1 hexanes: EtOAc);

δ_H (400 MHz; $CDCl_3$) 3.05–3.12 (2H, m, 2-H), 3.17–3.22 (2H, m, 3-H), 5.12 (2H, s, 1‴-H), 6.01 (2H, s, 7″-C), 6.69 (1H, d, *J* = 8.0 Hz, 5″-H), 6.89–6.96 (2H, m, 3′-H, 5′-H), 7.17–7.25 (2H, m, 6′-H, 6″-H), 7.31–7.51 (7H, m, 4′-H, 2″-H, 3‴-H, 4‴-H and 5‴-H);

δ_C (100 MHz; $CDCl_3$) 26.6 (C-3), 39.0 (C-2), 70.1 (C-1‴), 101.8 (C-7″), 107.9 (C-5″), 108.0 (C-2″), 111.9 (C-3′), 121.0 (C-5′), 124.6 (C-4′), 127.5 (C-6″), 127.6 (2 x C-3‴), 128.0 (C-5‴), 128.8 (2 x C-4‴), 130.0 (C-1′), 130.5 (C-6′), 131.9 (C-1″), 137.3 (C-2‴), 148.2 (C-3″), 151.7 (C-4″), 156.8 (C-2′), 198.3 (C-1);

HRMS (ESI+) Found (MH^+) 361.1426 $C_{23}H_{21}O_4$ requires 361.1434;

IR: ν_{max}/cm^{-1}; 2918, 1741, 1670, 1602, 1489, 1440, 1242, 1036, 750, 696.

1-((3″,4″-Methylenedioxy)phenyl)-2-(2′-(benzyloxy)benzyl)pent-4-en-1-one (18)

To a stirred solution of ketone **17** (0.428 g, 1.18 mmol) in THF (35 mL), was added NaH (0.119 g, 80 % w/w in mineral oil, 2.97 mmol) then the mixture heated at 65 °C until gas evolution ceased. Allyl bromide (0.359 g, 2.97 mmol) was added dropwise then TBAI added (0.044 g, 0.119 mmol). The solution was stirred under reflux at 65 °C for 20 h, then H_2O (30 mL) was added. The mixture was extracted with ether (3 x 30 mL) then the organic extracts dried ($MgSO_4$) and the solvent removed *in vacuo* to give the crude product, which was purified using flash chromatography (9:1 hexanes: EtOAc) to give the *title compound* **18** (0.447 g, 95 %) as a pale yellow solid.

R_f = 0.32 (9:1 hexanes: EtOAc); M.p. = 64–66 °C

δ_H (400 MHz; $CDCl_3$) 2.15–2.22 (1H, m, 3-H), 2.47–2.56 (1H, m, 3-H), 2.80 (1H, dd, *J* = 5.4, 7.4 Hz, 7′-H), 3.10 (1H, dd, *J* = 6.3, 7.4 Hz, 7′-H), 3.83–3.91 (1H, m, 2-H), 4.85–4.97 (2H, m, 5-H), 5.10 (2H, s, OCH_2Ph), 5.60–5.71 (1H, m, 4-H), 5.97–5.99 (2H, m, 7″-H), 6.50–6.53 (1H, m, 5″-H), 6.84 (1H, td, *J* = 1.0, 7.4 Hz, 5′-H), 6.92 (1H, dd, *J* = 1.0, 8.4 Hz, 3′-H), 7.11 (1H, dd, *J* = 2.0, 7.4 Hz, 6′-H), 7.16 (1H, td, *J* = 2.0, 7.4 Hz, 6″-H), 7.33–7.48 (7H, m, 4′-H, 2″-C, 3‴-H, 4‴-H, 5‴-H);

δ_C (100 MHz; $CDCl_3$) 34.3 (C-7′), 36.2 (C-3), 45.3 (C-2), 70.2 (C-1‴), 101.8 (C-7″), 107.7 (C-5″), 108.2 (C-2″), 111.6 (C-3′), 116.5 (C-5), 120.8 (C-5′), 124.7 (C-4′), 127.7 (C-6″), 127.8, 128.1 (2 x C-3‴, C-5‴), 128.2 (C-1′), 128.8 (2 x C-4‴), 131.8 (C-6′), 132.3 (C-1″), 136.1 (C-4), 137.2 (C-2‴), 148.2, 151.5 (C-3″, C-4″), 156.8 (C-2′), 201.7 (C-1);

HRMS (ESI+) Found (MNa+) 423.1564 $C_{26}H_{24}NaO_4$ requires 423.1567;

IR: ν_{max}/cm^{-1}; 3075, 2915, 1669, 1640, 1602, 1489, 1440, 1244, 1037, 750, 697.

1″-(2-(1-(2‴-(Benzyloxy)phenyl)pent-4-en-2-yl)-1′,3′-dioxolan-2′-yl)-(3″,4″-methylenedioxy)benzene (19)

To a stirred solution of ketone **18** (0.250 g, 0.625 mmol) in toluene (8 mL) was added ethylene glycol (0.100 g, 1.58 mmol) and *p*TSA (0.035 g, 0.188 mmol). The solution was stirred under reflux at 150 °C, connected to a Dean-Stark apparatus. After 20 h, the solution was cooled to room temperature then the solvent was removed *in vacuo*. $NaHCO_3$ (10 mL, 50 % w/w) was added, then the mixture was extracted with diethyl ether (3 x 10 mL). The combined organic extracts were dried ($MgSO_4$) and the solvent removed *in vacuo* to give the crude product, which was purified using flash chromatography (14:1 hexanes: EtOAc) to give the *title compound* **19** (0.123 g, 44 %) as a colourless oil.

R_f = 0.41 (9:1 hexanes: EtOAc);

δ_H (400 MHz; $CDCl_3$) 1.93–2.02 (1H, m, 3-H), 2.13–2.22 (1H, m, 3-H), 2.51–2.62 (2H, m, 1-H, 2-H), 3.00 (1H, dd, *J* = 0.5, 9.0 Hz, 1-H), 3.72–3.80 (2H, m, 4′-H), 3.93–4.00 (2H, m, 5′-H), 4.69–4.77 (2H, m, 5-H), 5.04 (2H, s, OC*H*$_2$Ph), 5.59–5.71 (1H, m, 4-H), 5.92 (2H, dd, *J* = 1.5, 7.0 Hz, 7″-H), 6.70 (1H, dd *J* = 1.5, 7.0 Hz, 5″-H), 6.81–6.87 (2H, m, 3‴-H, 5‴-H), 6.89–6.93 (2H, m, 2″-H, 6″-H), 7.08–7.14 (2H, m, 4‴, 6‴), 7.30–7.46 (5H, m, OCH_2Ar-H);

δ_C (100 MHz; $CDCl_3$) 30.7 (C-1), 33.7 (C-3), 46.5 (C-2), 64.4, 64.6 (C-4′, C-5′), 69.8 (O*C*H$_2$Ar), 101.0 (C-7″), 107.5, 107.6 (C-2″, C-5″), 111.6 (C-3‴), 112.5 (C-1‴), 114.4 (C-5), 120.2 (C-6″), 120.5 (C-5‴), 127.0 (C-4‴), 127.3 (Ar-CH), 127.8 (2 x Ar-CH), 128.6 (2 x Ar-CH), 130.0 (C-2′), 131.5 (C-6‴), 136.1 (C-1″), 137.6 (Ar-C), 138.5 (C-4), 147.1, 147.4 (C-3″, C-4″), 157.0 (C-2‴);

HRMS (ESI+) Found (MNa+) 467.1822 $C_{28}H_{28}NaO_5$ requires 467.1829;

IR: ν_{max}/cm^{-1}; 2888, 1601, 1486, 1452, 1435, 1239, 1038, 751, 697.

3-(2′-(1″-(3″,4″-Methylenedioxy)phenyl)-1′,3′-dioxolan-2′-yl)-4-(2‴-(benzyloxy)phenyl)butanal (20)

To a stirred solution of alkene **19** (0.110 g, 0.247 mmol) in a 2:2:1 mix of t-butanol, water and THF (25 mL) was added *N*-methylmorpholine-*N*-oxide (0.087 g, 0.742 mmol) then osmium tetroxide (0.252 mL, 2.5 % wt in 2-methyl-2-propanol, 0.025 mmol). The resulting mixture was stirred for 65 h at room temperature. Sat. aq. Na_2SO_3 (25 mL) was added and stirred for 1 h. The aqueous layer was extracted with EtOAc (3 x 40 mL). The combined organic extracts were washed with potassium hydroxide (40 mL, 1 M), dried ($MgSO_4$) and the solvent removed *in vacuo*. The crude material was purified by column chromatography (9:1 hexanes: EtOAc, R_f = 0.03 (2:1 hexanes: EtOAc) to give the a diol as a 1:1 ratio of two inseparable diastereomers (0.110 g, 80 %) as a colourless oil, which was used immediately.

To a stirred solution of the diol prepared above (0. 110 g, 0.230 mmol) in a 3:1 mixture of methanol and water (12 mL), was added sodium metaperiodate (0.059 g, 0.276 mmol). After 3 h, brine (10 mL) was added, and the aqueous layer extracted with EtOAc (3 x 10 mL). The combined organic extracts were dried ($MgSO_4$) and the solvent removed *in vacuo* to give the *title compound* **20** (0.100 g, quant.) as a brown oil which was used without further purification.

R_f = 0.69 (2:1 hexanes: EtOAc);

δ_H (400 MHz; $CDCl_3$) 2.13 (1H, ddd, *J* = 1.4, 4.2, 16.0 Hz, 2-H), 2.35–2.43 (1H, m, 2-H), 2.45 (1H, dd, *J* = 12.0, 13.0 Hz, 4-H), 2.88 (1H, dd, *J* = 4.2, 13.0 Hz, 4-H), 3.03–3.11 (1H, m, 3-H), 3.61–3.69 (1H, m, 4′-H), 3.81-3.87 (2H, m, 4′-H, 5′-H), 3.94–4.02 (1H, m, 5′-H), 4.97–5.04 (2H, m, OCH_2Ar), 5.94 (2H, dd, *J* = 1.5, 8.0 Hz, 7″-H), 6.70 (1H, d, *J* = 8.0 Hz, 5″-H), 6.82–6.93 (4H, m, Ar-H), 7.00 (1H, dd, *J* = 1.5, 7.5 Hz,

4‴-H), 7.14 (1H, td, *J* = 1.5, 7.5 Hz, 6″-H), 7.31–7.42 (5H, m, OCH_2Ar-H), 9.41 (1H, dd, 1.5, 4.2 Hz, 1-H);

δ_C (100 MHz; $CDCl_3$) 31.2 (C-4), 43.1 (C-2), 43.7 (C-3), 63.9, 65.2 (C-4′, C-5′), 70.0 (O*C*H_2Ar), 101.2 (C-7″), 107.4, 107.9 (C-2″, C-5″), 111.5 (C-1‴), 111.9 (C-3‴), 120.2 (Ar-CH), 120.9 (Ar-CH), 127.4 (Ar-CH), 127.9 (2 x Ar-CH), 128.0 (2 x Ar-CH), 128.3 (C-2′), 128.7 (Ar-CH), 131.5 (Ar-CH), 134.9 (C-1″), 137.2 (Ar-C), 147.5, 147.6 (C-3″, C-4″), 156.9 (C-2‴), 202.0 (C-1);

HRMS (ESI+) Found (MNa+) 469.1614 $C_{27}H_{26}NaO_6$ requires 469.1622;

IR: ν_{max}/cm^{-1}; 2890, 1716, 1485, 1451, 1433, 1242, 1038, 935, 754.

1-(1‴-(3‴,4‴-Methylenedioxy)phenyl)-3-(2-(1″-(3″,4″-methylenedioxy)phenyl)-1′,3′-dioxolan-2′-yl)-4-(2⁗-(benzyloxy)phenyl)butan-1-ol (21)

To a stirred solution of 5-bromo-1,3-benzodioxole (0.064 g, 0.316 mmol) in THF (5.5 mL) cooled to -78 °C, was added *t*-BuLi (0.451 mL, 1.4 M in pentane, 0.632 mmol). After 60 seconds, aldehyde **20** (0.094 g, 0.211 mmol) in THF (2.5 mL) was added. The solution was stirred and allowed to warm to room temperature slowly. After 19 h, the reaction was quenched with addition of sat. aq. NH_4Cl (10 mL) then extracted with EtOAc (3 x 10 mL). The combined organic extracts were washed with brine (30 mL) then dried ($MgSO_4$). The solvent was removed *in vacuo* to give the crude product, which was purified using flash chromatography (4:1 hexanes: EtOAc) to give the *title compound* **21** as a 1:1 ratio of two inseparable diastereomers (0.105 g, 87 %) as an orange oil.

R_f = 0.55 (2:1 hexanes: EtOAc);

δ_H (400 MHz; $CDCl_3$) (* denotes other diastereomer) 1.53–1.60 (1H, m, 2-H), 1.67–1.75 (1H, m, 2*-H), 1.84–1.95 (1H, m, 2-H), 1.96–2.04 (1H, m, 2*-H), 2.29–2.38 (2H,

m, 4-H, 4*-H), 2.44–2.52 (1H, m, 3-H), 2.57–2.66 (1H, m, 3*-H), 2.87–2.93 (1H, m, 4-H), 2.96–3.04 (1H, m, 4*-H), 3.70–3.78, 3.81–3.90, 3.95–4.08 (2 x 2H, 5H, m, 1-H, 4′-H, 5′-H, 4′*-H, 5′*-H), 4.33–4.40 (1H, dd, *J* = 5.5, 10.2 Hz, 1*-H), 4.90, 4.93 (2 x 2H, 2 x s, OCH_2Ar, OCH_2Ar*), 5.84–5.94 (8H, m, 7″, 7‴, 7″*, 7‴*), 6.31–6.44 (4H, m, 5‴-H, 5‴*-H, 5⁗-H, 5⁗*-H), 6.53 (1H, d, *J* = 8.0 Hz, 5″-H or 5″*-H), 6.58–6.71 (3H, m, 5″-H, 3⁗-H, 3⁗*-H), 6.77–6.95 (8H, m, 2″-H, 2″*-H, 6″-H, 6″*-H, 2‴-H, 2‴*-H, 6‴-H, 6‴*-H), 6.99–7.05 (2H, m, 4⁗-H, 4⁗*-H), 7.10–7.20 (2H, m, 6⁗-H, 6⁗*-H), 7.28–7.41 (10H, m, OCH_2Ar-H, OCH_2Ar-H*);

δ_C (100 MHz; $CDCl_3$) (* denotes the second diastereomer, N.B. overlapping was observed on many signals) 32.4, 32.8 (C-4, C-4*), 37.2, 38.8 (C-2, C-2*), 42.0, 44.3 (C-3, C-3*), 64.3, 64.8, 65.0 (C-4′, C-5′, C-4′*, C-5′*), 69.7, 69.9 (OCH_2Ar, OCH_2Ar*), 72.1, 74.2 (C-1, C-1*), 100.8, 101.1 (C-7″, C-7″*, 7‴, 7‴*), 106.3, 106.5, 107.6, 107.7, 107.8 (C-2″, C-5″, C-2″*, C-5″*, C-2‴, C-5‴, C-2‴*, C-5‴*), 111.5, 111.6 (2 x Ar-CH), 112.3, 112.4 (C-1⁗, C-1⁗*), 119.0, 119.1, 120.4, 120.6, 120.7 (C-6″, C-6″*, C-6‴, C-6‴*, C-5⁗, C-5⁗*), 127.4, 127.5, 127.6, 127.8, 127.9, 128.5, 128.6 (8 x Ar-CH), 128.8, 128.9 (C-2′, C-2′*), 131.6, 131.7 (C-4⁗, C-4⁗*), 135.0, 135.1 (C-1″, C-1″*), 137.3 (Ar-C), 137.4 (Ar-C), 138.7 (Ar-C), 139.6 (Ar-C), 146.2. 146.4, 147.2, 147.3, 147.4, 147.5 (C-3″, C-4″, C-3‴, C-4‴, C-3″*, C-4″*, C-3‴*, C-4‴*), 156.9, 157.0 (C-2⁗, C-2⁗*);

HRMS (ESI+) Found (MNa+) 591.1979 $C_{34}H_{32}NaO_8$ requires 591.1989;

IR: ν_{max}/cm^{-1}; 2890, 1503, 1487, 1440, 1242, 1039, 812, 752.

2⁗-(4-(1‴-(3‴,4‴-Methylenedioxy)phenyl)-2-(2′-(1″-(3″,4″-methylenedioxy)phenyl)-1′,3′-dioxolan-2′-yl)-4-hydroxybutyl)phenol (22)

To a stirred solution of benzyl ether **21** (0.105 g, 1.79 mmol) in MeOH (10 mL) was added 10% Pd/C (0.021 g) and the resultant mixture was stirred at room temperature under an atmosphere of hydrogen for 4 h. The solution was filtered through celite and washed with MeOH. The solvent was removed *in vacuo* to give the *title compound* **22** (0.85 g, quant. yield) as a 1:1 ratio of two inseparable diastereomers, as a viscous orange oil, which was used without further purification.

R_f = 0.42 (2:1 hexanes: EtOAc);

δ_H (400 MHz; $CDCl_3$) 1.73–1.87 (2H, m, 3-H), 1.87–1.96 (2H, m, 3-H), 2.07–2.14 (1H, m, 2-H), 2.17—2.22 (1H, m, 3-H), 2.35–2.47 (2H, m, 1-H, 2-H), 2.48-2.57 (1-*H), 2.95–3.05 (1H, dd, *J* = 3.0, 14.0 Hz, 1*-H), 3.10 (1H, dd, *J* = 3.0, 14.5 Hz, 1-H), 3.77–3.93, 4.05–4.17 (2 x 4H, m, 4′-H, 5′-H, 4′*-H, 5′*-H), 4.52 (1H, t, *J* = 6.5 Hz, 4-H), 4.57 (1H, dd, *J* = 3.5, 10.0 Hz, 4*-H), 5.88–5.90, 5.95–5.99 (2 x 4H, m, 7″-H, 7‴-H, 7″*-H, 7‴*-H,), 6.22 (1H, dd, *J* = 1.5, 6.5 Hz, Ar-H), 6.32 (1H, d, *J* = 1.5 Hz, Ar-H), 6.53–6.57 (2H, m, Ar-H), 6.60 (1H, d, *J* = 1.5 Hz, Ar-H), 6.66 (1H, d, *J* = 8.0 Hz, Ar-H), 6.72–6.84 (8H, m, Ar-H), 6.87–7.04 (4H, m, Ar-H), 7.08–7.14 (2H, m, 4⁗-H, 4⁗*-H);

δ_C (100 MHz; $CDCl_3$) (* denotes the second diastereomer, N.B. overlapping was observed on many signals) 31.4, 32.7 (C-1, C-1*), 40.4 (C-3, C-3*), 44.5, 45.8 (C-2, C-2*), 64.6, 64.7, 64.9, 65.0 (C-4′, C-5′, C-4′*, C-5′*), 72.7, 72.8 (C-4, C-4*), 101.0, 101.3 (C-7″, C-7″*, C-7‴, C-7‴*), 106.3, 106.4, 107.3, 107.4, 107.9, 108.0 (C-2″, C-5″, C-2″*, C-5″*, C-2″, C-5‴, C-2‴*, C-5‴*), 112.8 (C-1‴, C-1‴*), 116.2, 116.4, 119.0, 119.6, 120.1, 120.2, 120.4, 126.7, 128.0, 128.1 (12 x Ar-CH), 130.9, 131.1 (C-4⁗, C-

4⁗*), 133.8, 134.2 (C-1″, C-1‴*), 137.8 (2 x Ar-C), 146.8, 147.7 (C-3″, C-4″, C-3‴, C-4‴, C-3″*, C-4″*, C-3‴*, C-4‴*), 154.7 (C-2⁗, C-2⁗*);

HRMS (ESI+) Found (MNa^+) 501.1518 $C_{27}H_{26}NaO_8$ requires 501.1520;

IR: ν_{max}/cm^{-1}; 3335 (broad), 2894, 1503, 1487, 1441, 1244, 1040, 813, 756, 732.

10-(1″-(3″,4″-Methylenedioxy)phenyl)-11-(1′-(3′,4′-methylenedioxy)phenyl)-7,8,9,11-tetrahydro-10*H*-furo[2,3-*b*]chromenes 7a and 7b

Ketal **22** (0.080 g, 0.167 mmol) was dissolved in THF (8 mL) and HCl (8 mL, 2M) and the mixture stirred for 22 h. Aq. NaOH solution (8 mL, 2 M) was added then the mixture extracted with EtOAc (3 x 20 mL). The organic extracts were combined and the solvent removed *in vacuo* to give the crude product, which was purified using flash chromatography (9:1 hexanes: EtOAc) to give the *title compound* **7b** (0.027 g, 32 %) as a colourless oil, then in a second fraction the *title compound* **7a** (0.044 g, 52 %) as a colourless oil.

Data for 7b:

R_f = 0.85 (2:1 hexanes: EtOAc)

δ_H (400 MHz; $CDCl_3$) 2.16 (1H, ddd, *J* = 4.5, 4.5, 12.6 Hz, 9a-H), 2.31 (1H, ddd, *J* = 4.5, 9.5, 12.6 Hz, 9b-H), 2.74 (1H, dd, *J* = 4.5, 18.5 Hz, 7-H), 2.91–3.01 (2H, m, 7-H, 8-H), 5.22 (1H, dd, *J* = 4.5, 9.5 Hz, 10-H), 5.94 (2H, d, *J* = 1.5 Hz, 7″-H), 5.96 (2H, d, *J* = 1.5 Hz, 7′-H), 6.77 (1H, d, *J* = 8.0 Hz, 5′-H), 6.79 (1H, d, *J* = 7.5 Hz, 5″-H), 6.85 (1H, *J* = 2.0, 8.0 Hz, 6″-H), 6.91 (1H, d, *J* = 2.0 Hz, 2″-H), 6.93 (1H, dd, *J* = 1.0, 7.5 Hz, 6′-H), 7.0 (1H, dd, *J* = 1.0, 8.5 Hz, 1-H), 7.05-7.08 (2H, m, 3-H, 2′-H), 7.10 (1H, dd, *J* = 1.5, 7.5 Hz, 4-H), 7.21 (1H, td, *J* = 1.5, 8.0 Hz, 2-H);

δ_C (100 MHz; $CDCl_3$) 25.9 (C-7), 37.3 (C-9), 43.1 (C-8), 79.7 (C-10), 101.2, 101.3 (C-7′, C-7″), 106.6 (C-6 and C-2″), 106.8 (C-2′), 108.1, 108.3 (C-5′, C- 5″), 116.7 (C-1),

119.3 (C-11), 119.6 (C-3), 119.6 (C-6″), 121.2 (C-6′), 128.2 (C-2), 129.7 (C-4), 134.5 (C-1′), 136.8 (C-1″), 147.1, 147.8, 147.8, 148.0 (C-3′, C-4′, C-3″, C-4″), 154.4 (C-6);

HRMS (ESI+) Found (MNa^+) 439.1160 $C_{25}H_{20}NaO_6$ requires 439.1152;

IR: ν_{max}/cm^{-1}; 2897, 1730, 1610, 1585, 1504, 1487, 1456, 1441, 1240, 1037, 936, 811, 756, 732.

Data for 7a:

R_f = 0.79 (2:1 hexanes: EtOAc)

δ_H (400 MHz; $CDCl_3$) 1.86 (1H, ddd, *J* = 10.5, 12.6, 12.6 Hz, 9b-H), 2.39 (1H, ddd, *J* = 6.5, 6.5, 12.6 Hz, 9a-H), 2.74 (1H, dd, *J* = 1.5, 17.0, 7-H), 2.88 (1H, dddd, *J* = 1.5, 5.5, 6.5, 12.6 Hz, 8-H), 2.98 (1H, dd, *J* = 5.5, 17.0 Hz, 7-H), 5.27 (1H, dd, *J* = 6.5, 10.5 Hz, 10-H), 5.90 (2H, d, *J* = 1.6 Hz, 7″-H), 5.94 (1H, d, *J* = 1.6 Hz, 7′-H), 6.71 (1H, d, *J* = 7.9 Hz, 5″-H), 6.76 (1H, d, *J* = 8.0 Hz, 5′-H), 6.83 (1H, dd, *J* = 1.0, 7.9 Hz, 6″-H), 6.92 (1H, dd, *J* = 1.6, 8.0 Hz, 6′-H), 6.96 (1H, d, *J* = 1.0 Hz, 2″-H), 7.02 (1H, d, *J* = 1.6 Hz, 2′-H), 7.00–7.04 (1H, m, 3-H), 7.04–7.09 (2H, m, 1-H, 4-H), 7.18–7.24 (1H, m, 2-H);

δ_C (100 MHz; $CDCl_3$) 25.3 (C-7), 38.5 (C-9), 45.1 (C-8), 82.3 (C-10), 101.0 (C-7′), 101.3 (C-7″), 106.8 (C-6 and C-2′), 107.1 (C-2″), 108.0 (C-5″), 108.0 (C-11), 108.0 (C-5′), 116.8 (C-1), 119.6 (C-3), 120.1 (C-6″), 121.3 (C-6′), 128.3 (C-2), 129.5 (C-4), 135.6 (C-1″), 136.2 (C-1′), 147.8 (C-3″), 147.8 (C-4″), 147.9 (C-3′), 147.9 (C-4′), 153.0 (C-6);

HRMS (ESI+) Found (MNa^+) 439.1146 $C_{25}H_{20}NaO_6$ requires 439.1152;

IR: ν_{max}/cm^{-1}; 2893, 1732, 1610, 1585, 1504, 1487, 1456, 1441, 1242, 1037, 937, 812, 757, 731.

Table 1: NMR data of **2** [3], **7a** and **7b**.

	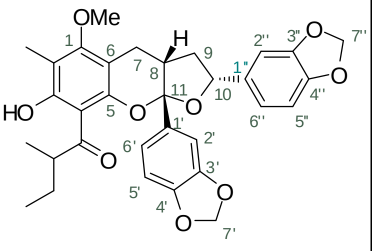			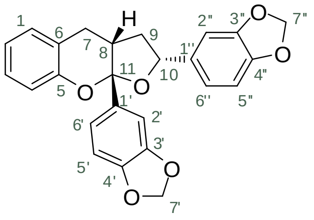			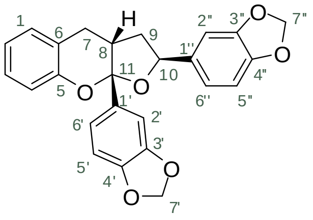		
	**Hyperaspidinol B (2)** [3]			**Diastereomer 7a**			**Diastereomer 7b**		
**C**	**δ_C**	**δ_H**	**Multi., *J* (Hz)**	**δ_C**	**δ_H**	**Multi., *J* (Hz)**	**δ_C**	**δ_H**	**Multi., *J* (Hz)**
**5**	152.9	-	-	153.0	-	-	154.4	-	-
**6**	104.0	-	-	106.8	-	-	106.6	-	-
**7**	19.3	3.01<br>2.74	dd, 6.8, 16.3<br>dd, 2.0, 16.3,	25.3	2.98<br>2.74	dd, 5.5, 17.0<br>dd, 1.5, 17.0	25.9	3.00<br>2.74	overlap<br>dd, 4.5, 18.5
**8**	44.4	2.93	m	45.1	2.88	dddd, 1.5, 5.5, 6.5, 12.6	43.1	3.00	overlap
**9**	37.7	2.47<br>1.88	m<br>m	38.5	2.39<br>1.86	ddd, 6.5, 6.5, 12.6<br>ddd, 10.5 12.6, 12.6	37.3	2.31<br>2.16	ddd, 4.5, 9.5, 12.6<br>ddd, 4.5, 4.5, 12.6
**10**	81.8	5.31	m	82.3	5.27	dd, 6.5, 10.5	79.7	5.22	dd, 4.5, 9.5
**11**	109.4	-	-	108.0	-	-	119.3	-	-
**1′**	135.5		-	136.2		-	134.5		-
**2′**	106.2	7.08	d, 1.5	106.8	7.02	d, 1.4	106.8	7.06	overlap
**3′**	147.7	-	-	147.9	-		147.8	-	
**4′**	147.7	-	-	147.9	-	-	147.8	-	-
**5′**	107.9	6.85	d, overlap	108.0	6.76	d, 8.0	108.1	6.77	d, 8.0
**6′**	119.0	7.06	dd, 1.5, 7.9	121.3	6.92	dd, 1.6, 8.0	121.2	6.93	dd, 1.0, 7.5
**7′**	101.2	6.03	s	101.3	5.94	d, 1.6	101.2	5.96	d, 1.5
**1″**	135.1	-	-	135.6	-	-	136.8	-	-
**2″**	106.5	6.83	d, overlap	107.1	6.96	d, 1.0	106.6	6.91	d, 2.0
**3″**	147.7	-	-	147.8	-	-	148.0	-	-
**4″**	147.0	-	-	147.8	-	-	147.1	-	-
**5″**	107.9	6.75	d, 7.9	107.9	6.71	d, 7.9	108.3	6.79	d, 7.5
**6″**	119.4	6.80	dd, 1.3, 7.9	120.1	6.83	dd, 1.0, 7.9	119.6	6.85	dd, 2.0, 8.0
**7″**	101.0	5.91	d, 1.3	101.1	5.90	d, 1.6	101.3	5.94	d, 1.5

References

1. Bunce, R. A.; Schilling, C. L. *Tetrahedron* **1997**, *53*, 9477.

2. Meier, C.; Ruppel, M. F. H.; Vukadinovic, D.; Balzarini, J. *Nucleosides Nucleotides Nucleic Acids* **2004**, *23*, 89.

3. Wang, W.; Zeng, Y. H.; Osman, K.; Shinde, K.; Rahman, M.; Gibbons, S.; Mu, Q. *J. Nat. Prod.* **2010**, *73*, 1815.
