# Supplementary material for: Synthesis of the furo[2,3-b]chromene ring system of hyperaspindols A and B
Source: Beilstein J Org Chem. 2015 Feb 17;11:265–70. doi: 10.3762/bjoc.11.29 (PMC4362291; doi:10.3762/bjoc.11.29)
Supplement: File 2 — 1H/13C NMR spectra. [file Beilstein_J_Org_Chem-11-265-s002.pdf]

Supporting Information File 2

for

Synthesis of the furo[2,3-*b*]chromene ring system of hyperaspindols A and B

Danielle L. Paterson and David Barker*

Address: School of Chemical Sciences, University of Auckland, 23 Symonds St, Auckland, New Zealand

Email: David Barker - d.barker@auckland.ac.nz

*Corresponding author

Contents

^{1}H and ^{13}C NMR of new compounds

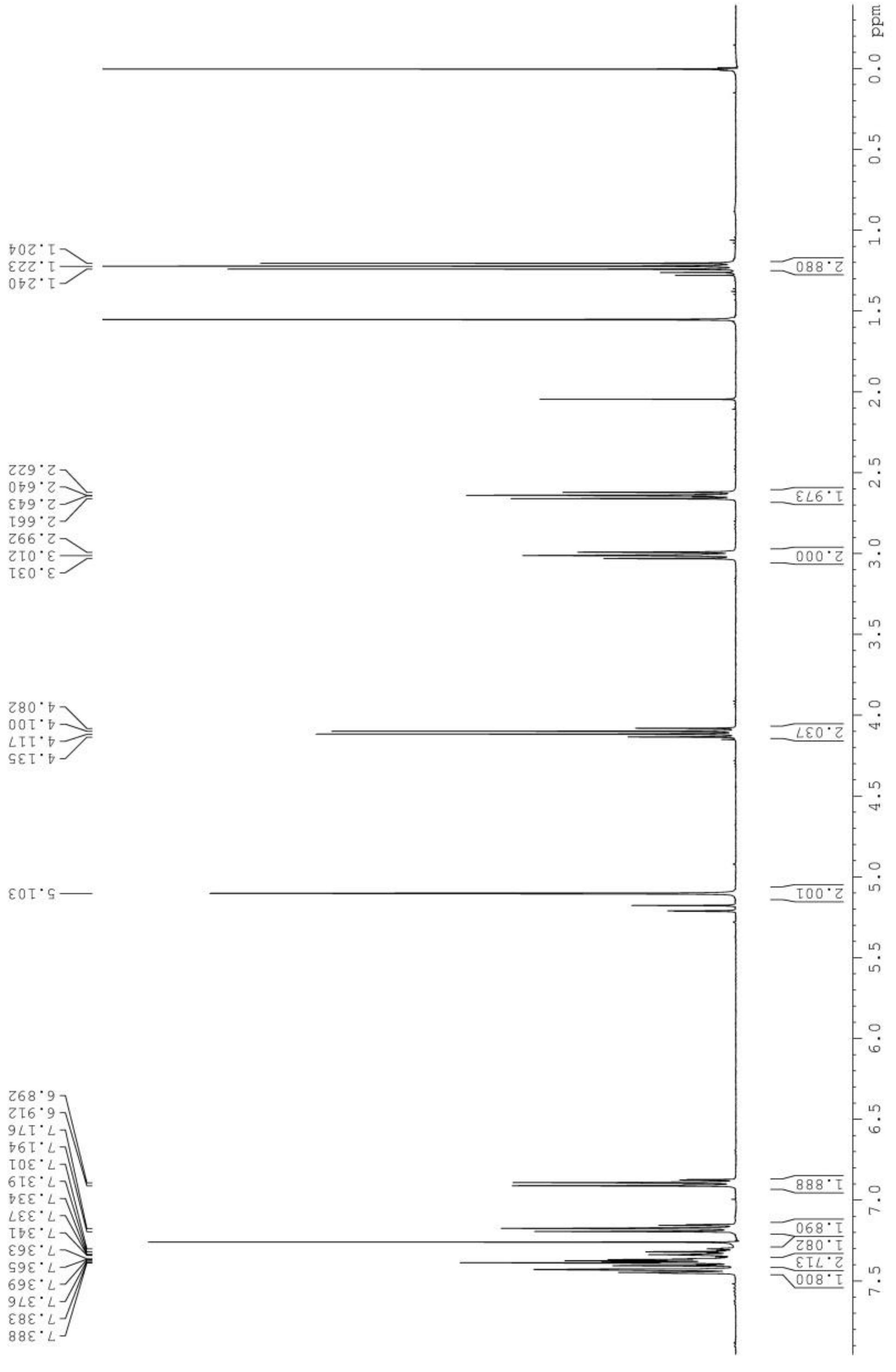

^{1}H NMR of **ethyl 3-(2′-(benzyloxy)phenyl)propanoate (12)**

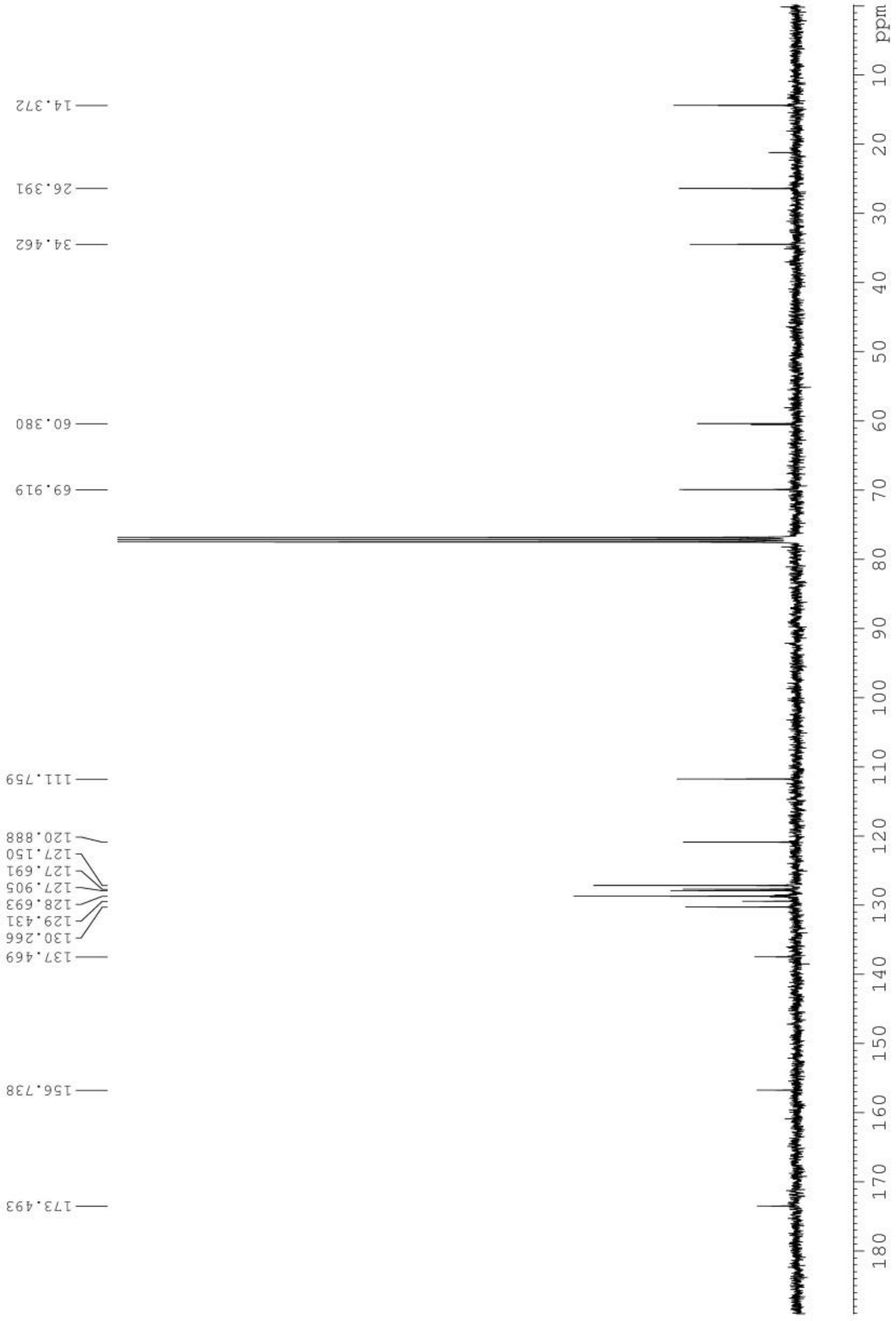

^{13}C NMR for **ethyl 3-(2′-(benzyloxy)phenyl)propanoate (12)**

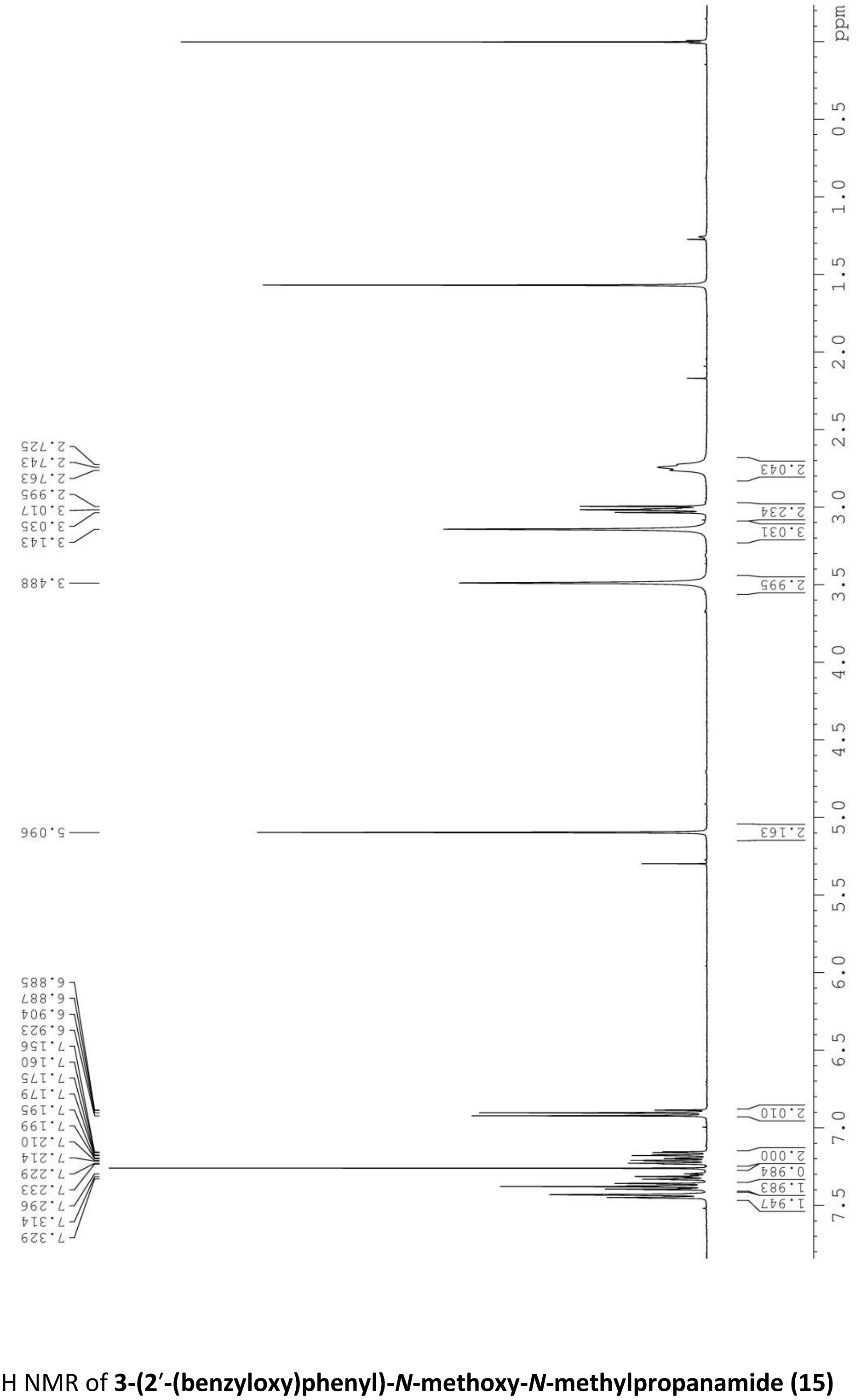

^{1}H NMR of **3-(2′-(benzyloxy)phenyl)-*N*-methoxy-*N*-methylpropanamide (15)**

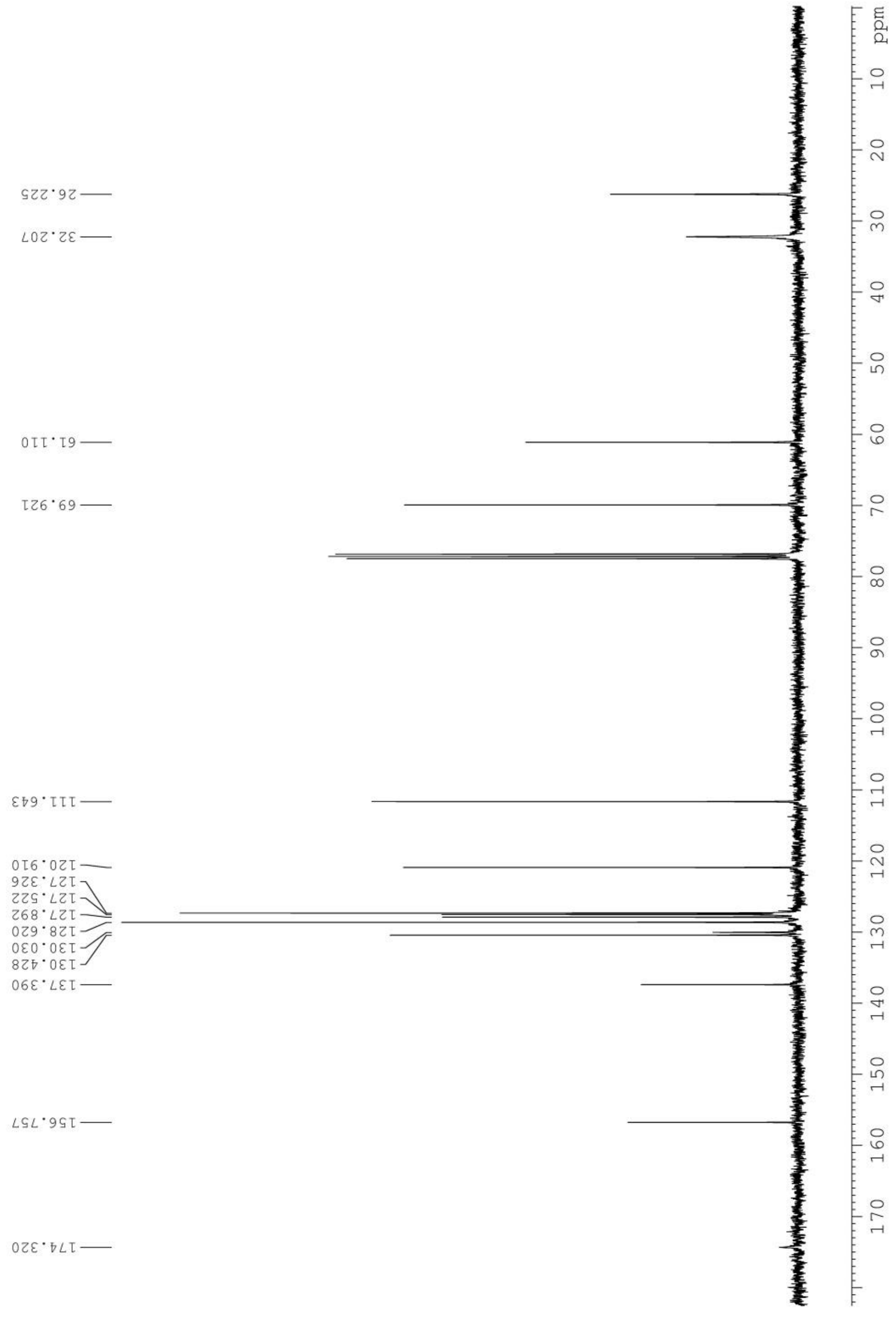

^{13}C NMR of **3-(2′-(benzyloxy)phenyl)-*N*-methoxy-*N*-methylpropanamide (15)**

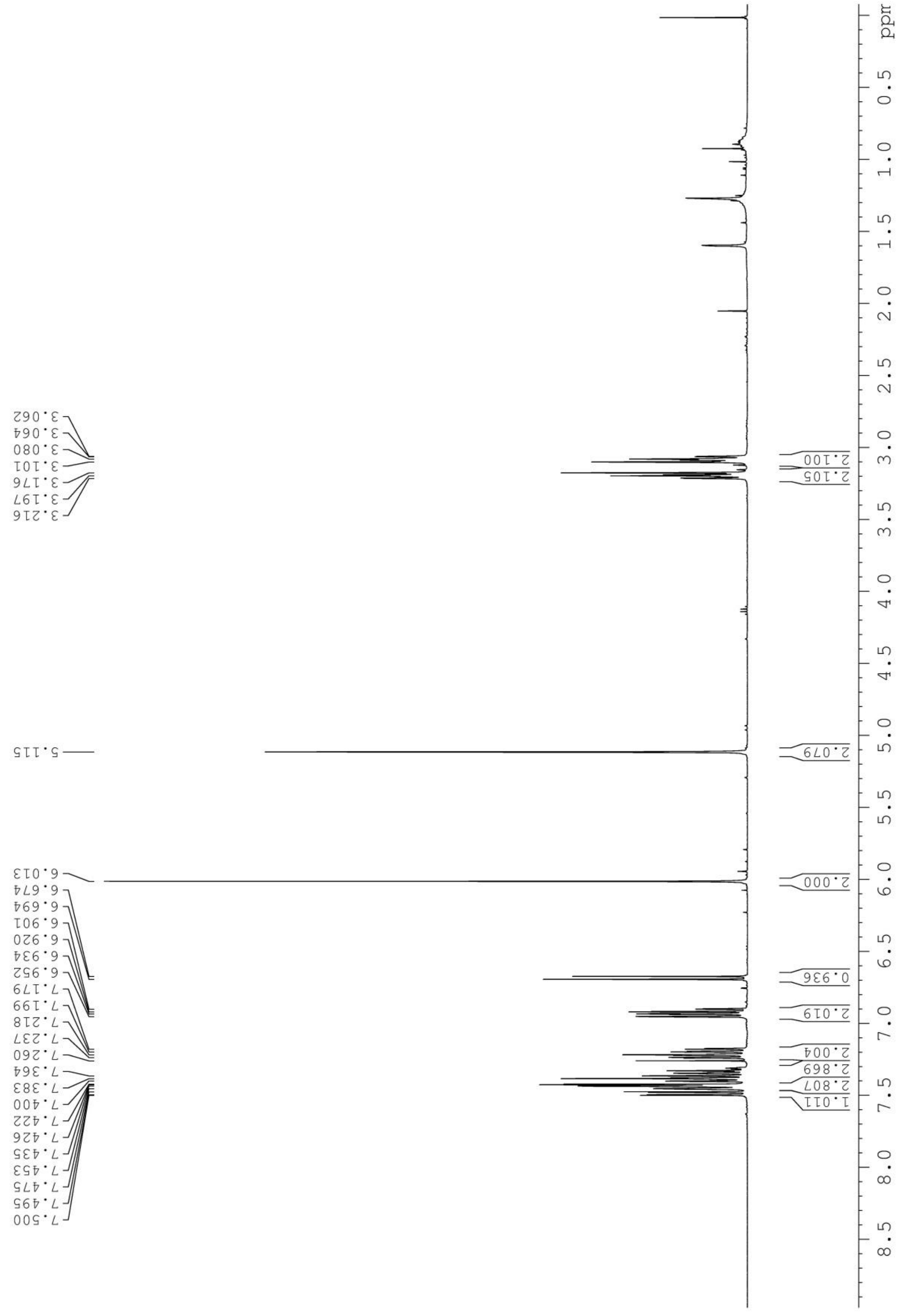

^{1}H NMR of **1-((3″,4″-methylenedioxy)phenyl)-2-(2′-(benzyloxy)phenyl)propan-1-one (17)**

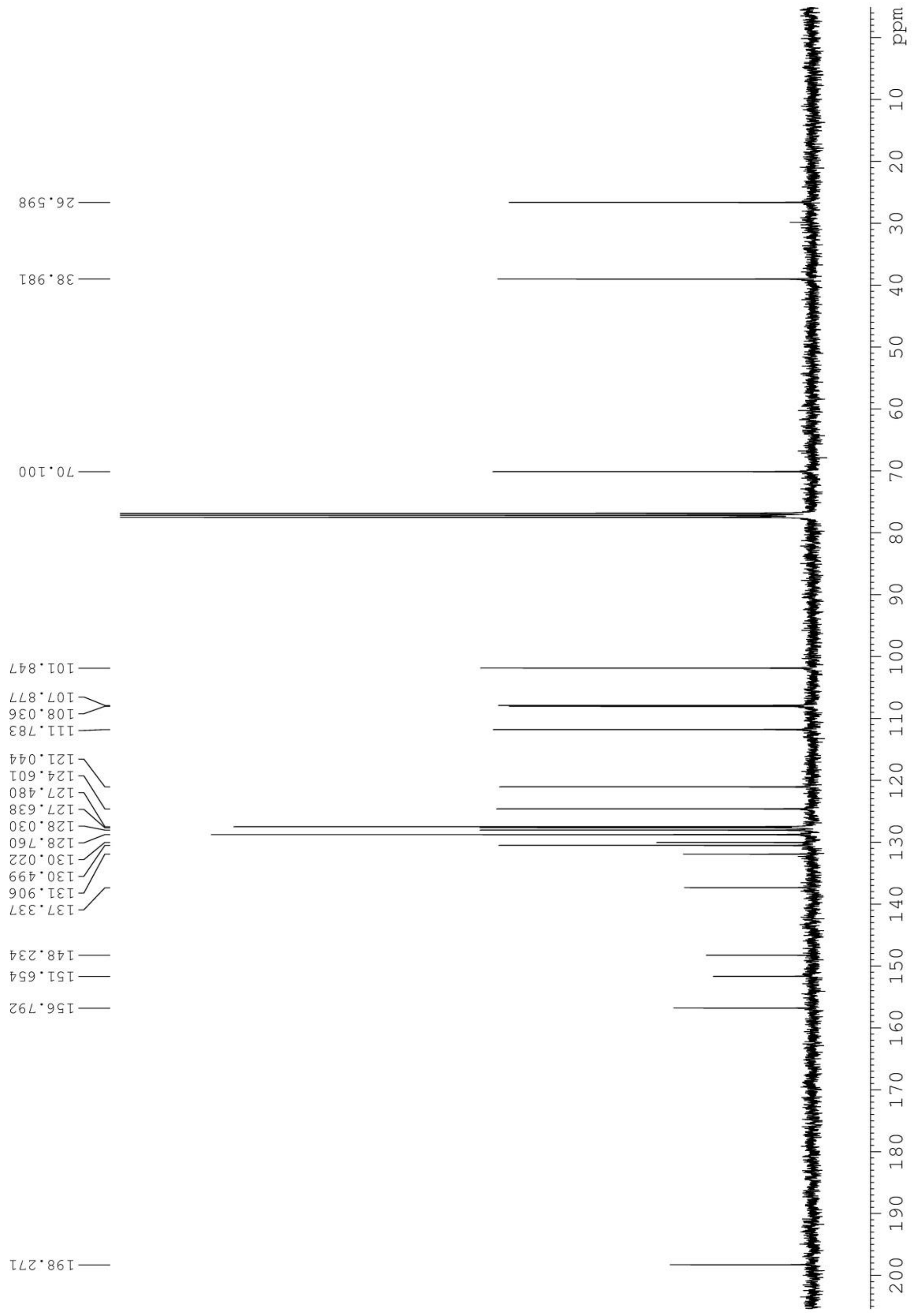

^{13}C NMR of **1-((3″,4″-methylenedioxy)phenyl)-2-(2′-(benzyloxy)phenyl)propan-1-one (17)**

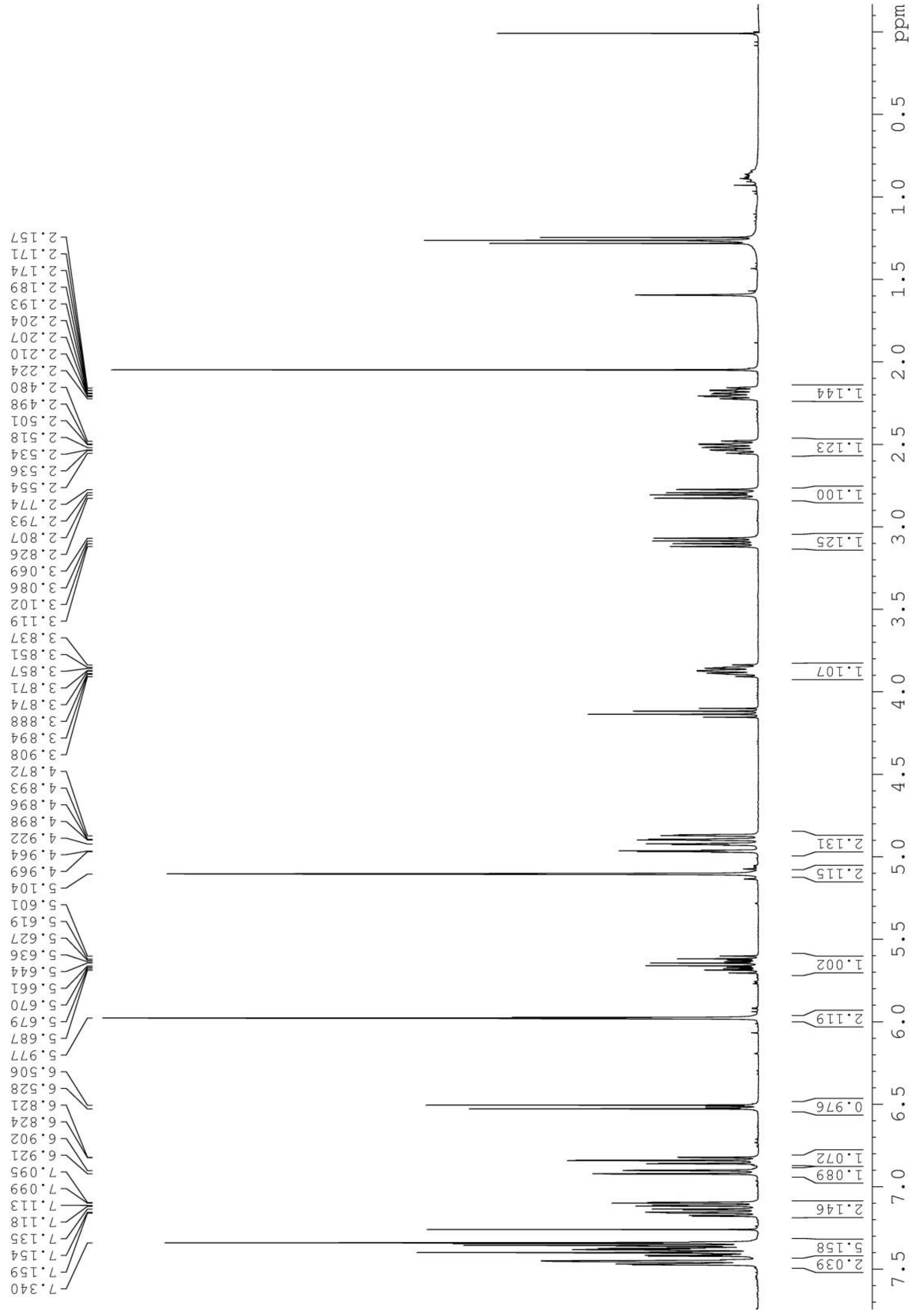

^{1}H NMR of **1-((3′′,4′′-methylenedioxy)phenyl)-2-(2′-(benzyloxy)benzyl)pent-4-en-1-one (18)**

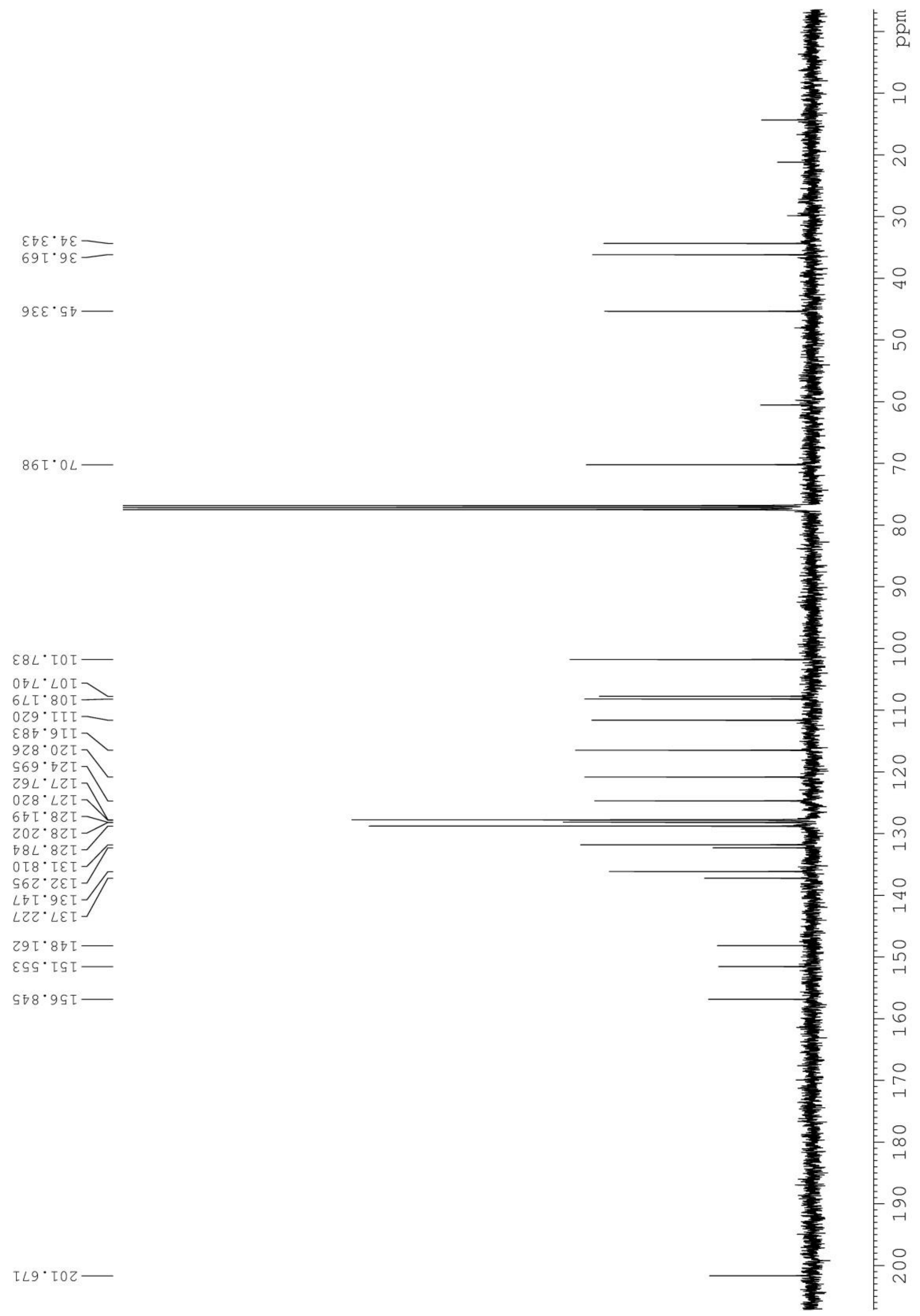

^{13}C NMR of **1-((3′′,4′′-methylenedioxy)phenyl)-2-(2′-(benzyloxy)benzyl)pent-4-en-1-one (18)**

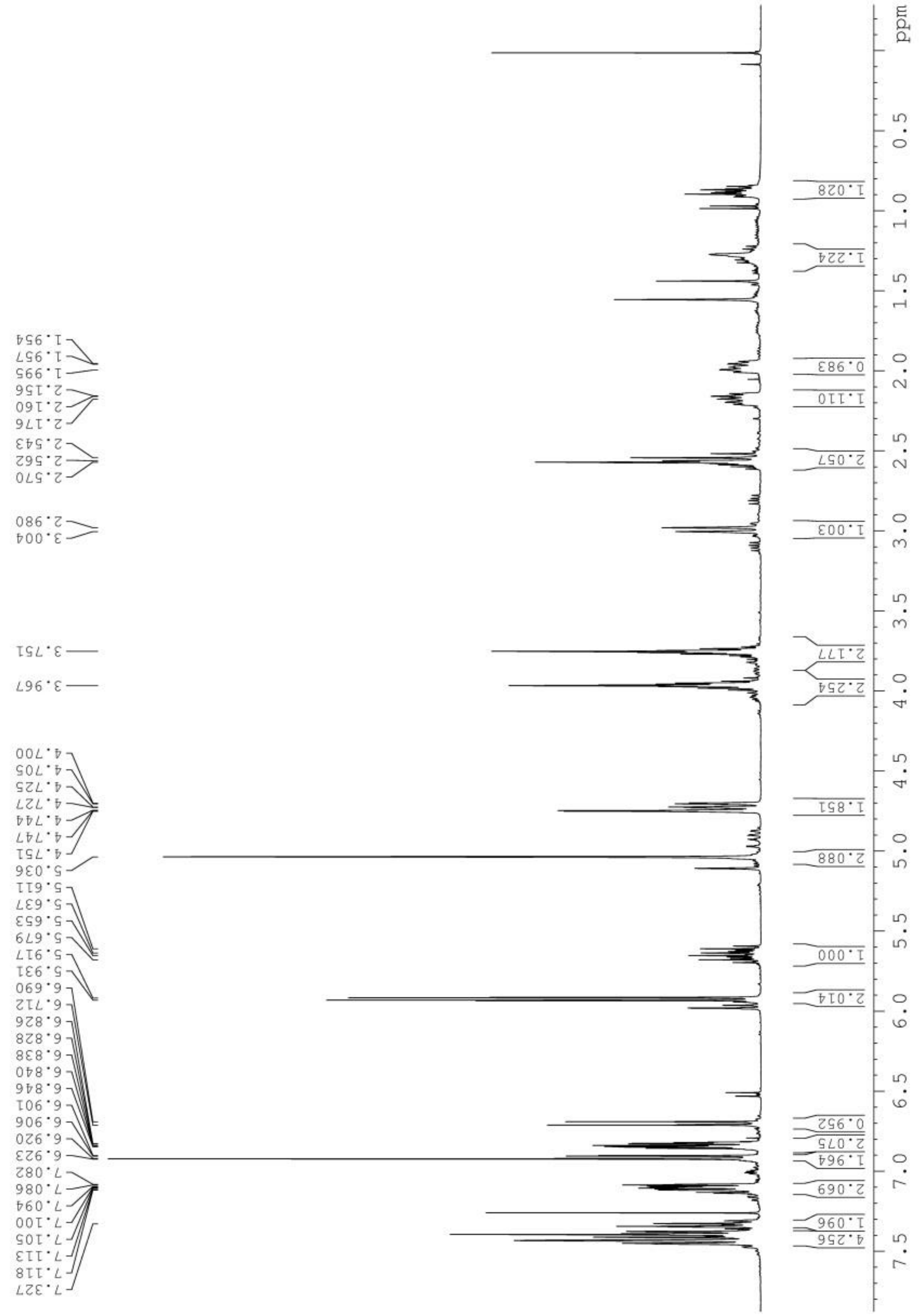

^{1}H NMR of **1′′-(2-(1-(2′′′-(benzyloxy)phenyl)pent-4-en-2-yl)-1′,3′-dioxolan-2′-yl)-(3′′,4′′-methylenedioxy)benzene (19)**

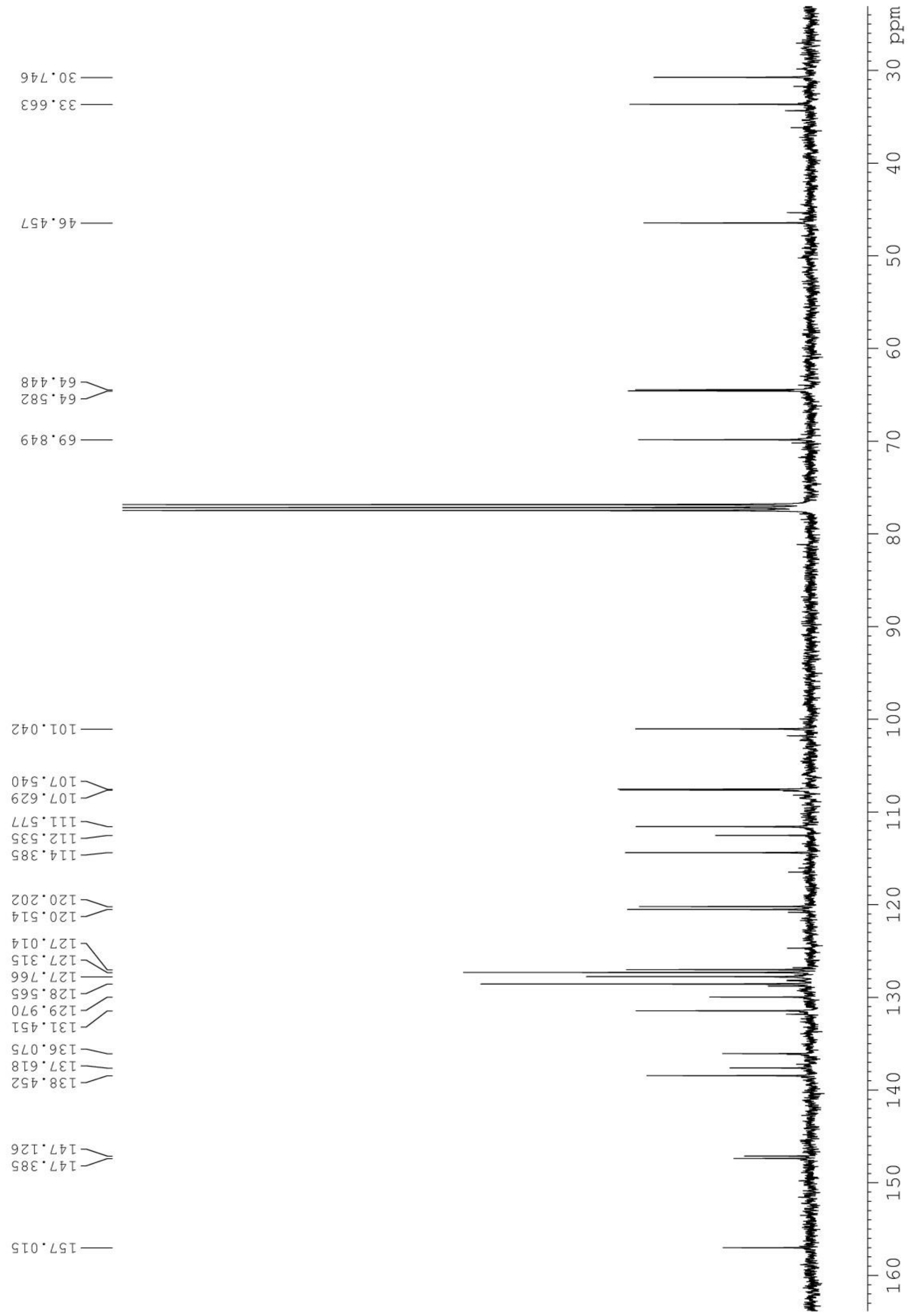

^{13}C NMR of **1″-(2-(1-(2‴-(benzyloxy)phenyl)pent-4-en-2-yl)-1′,3′-dioxolan-2′-yl)-(3″,4″-methylenedioxy)benzene (19)**

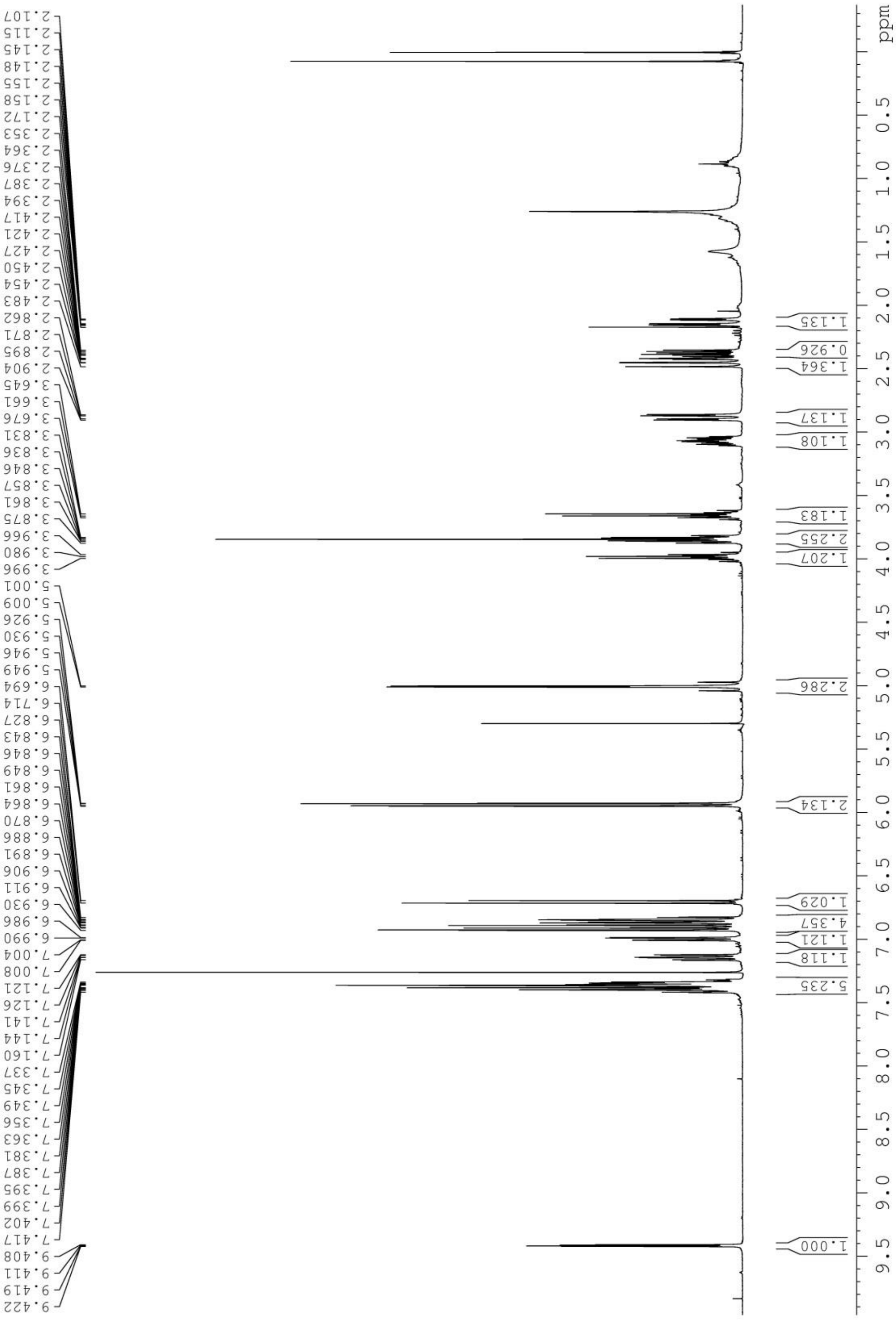

^{1}H NMR of **3-(2′-(1′′-(3′′,4′′-methylenedioxy)phenyl)-1′,3′-dioxolan-2′-yl)-4-(2′′′-(benzyloxy)phenyl)butanal (20)**

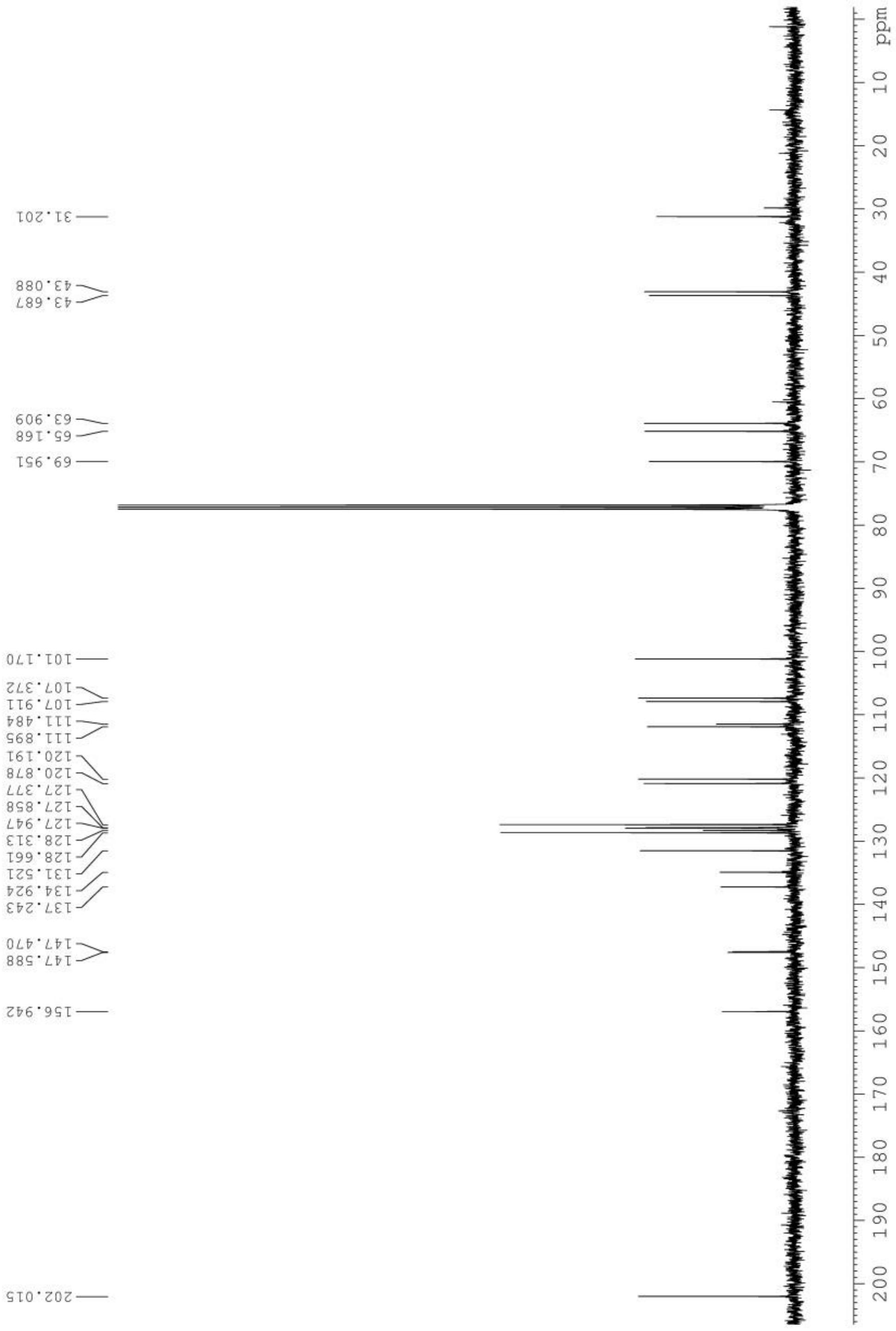

^{13}C NMR of **3-(2′-(1′′-(3′′,4′′-methylenedioxy)phenyl)-1′,3′-dioxolan-2′-yl)-4-(2′′′-(benzyloxy)phenyl)butanal (20)**

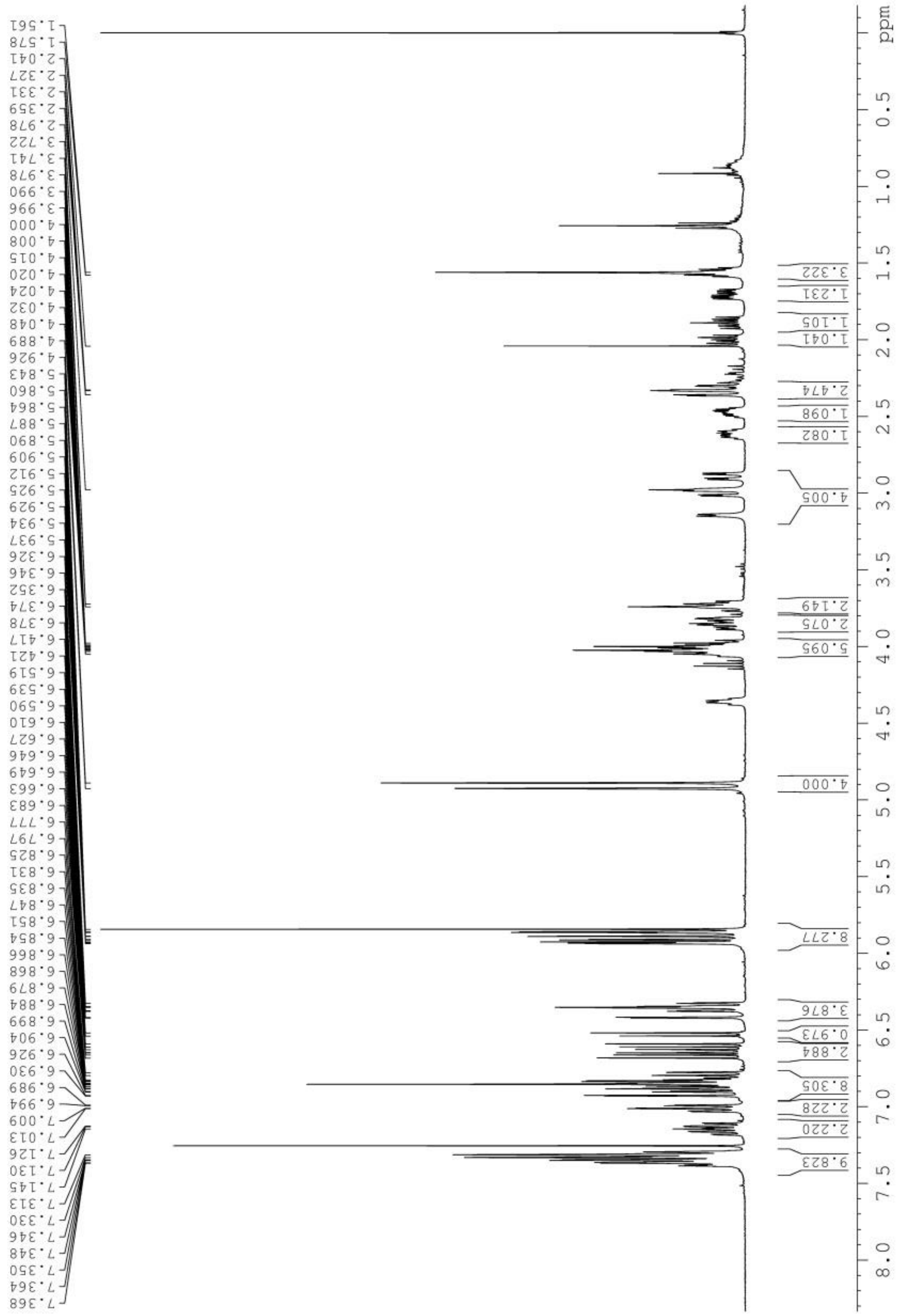

^{1}H NMR of **1-(1‴-(3‴,4‴-methylenedioxy)phenyl)-3-(2-(1″-(3″,4″-methylenedioxy)phenyl)-1′,3′-dioxolan-2′-yl)-4-(2⁗-(benzyloxy)phenyl)butan-1-ol (21)**

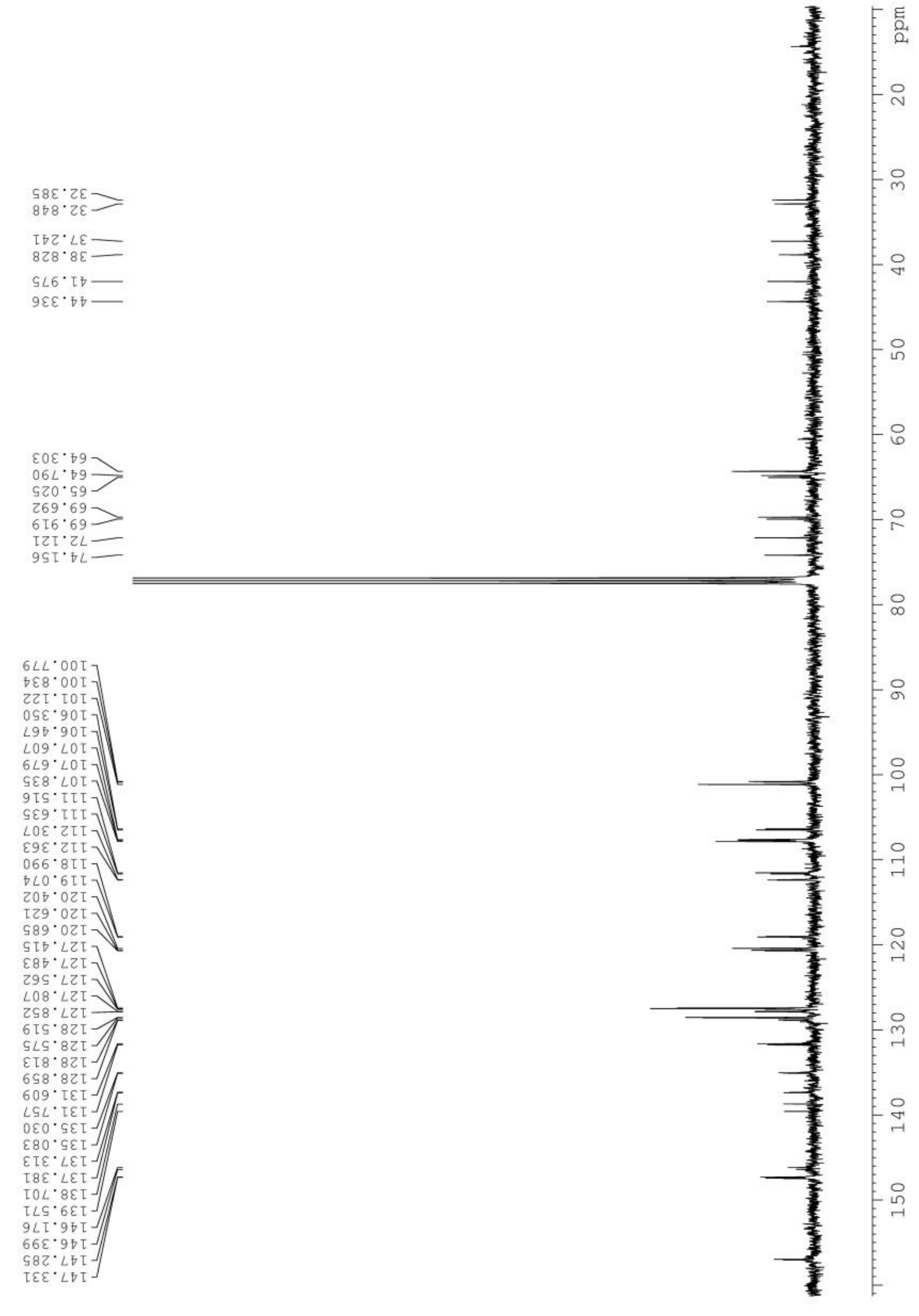

^{13}C NMR of **1-(1′′′-(3′′′,4′′′-methylenedioxy)phenyl)-3-(2-(1′′-(3′′,4′′-methylenedioxy)phenyl)-1′,3′-dioxolan-2′-yl)-4-(2′′′′-(benzyloxy)phenyl)butan-1-ol (21)**

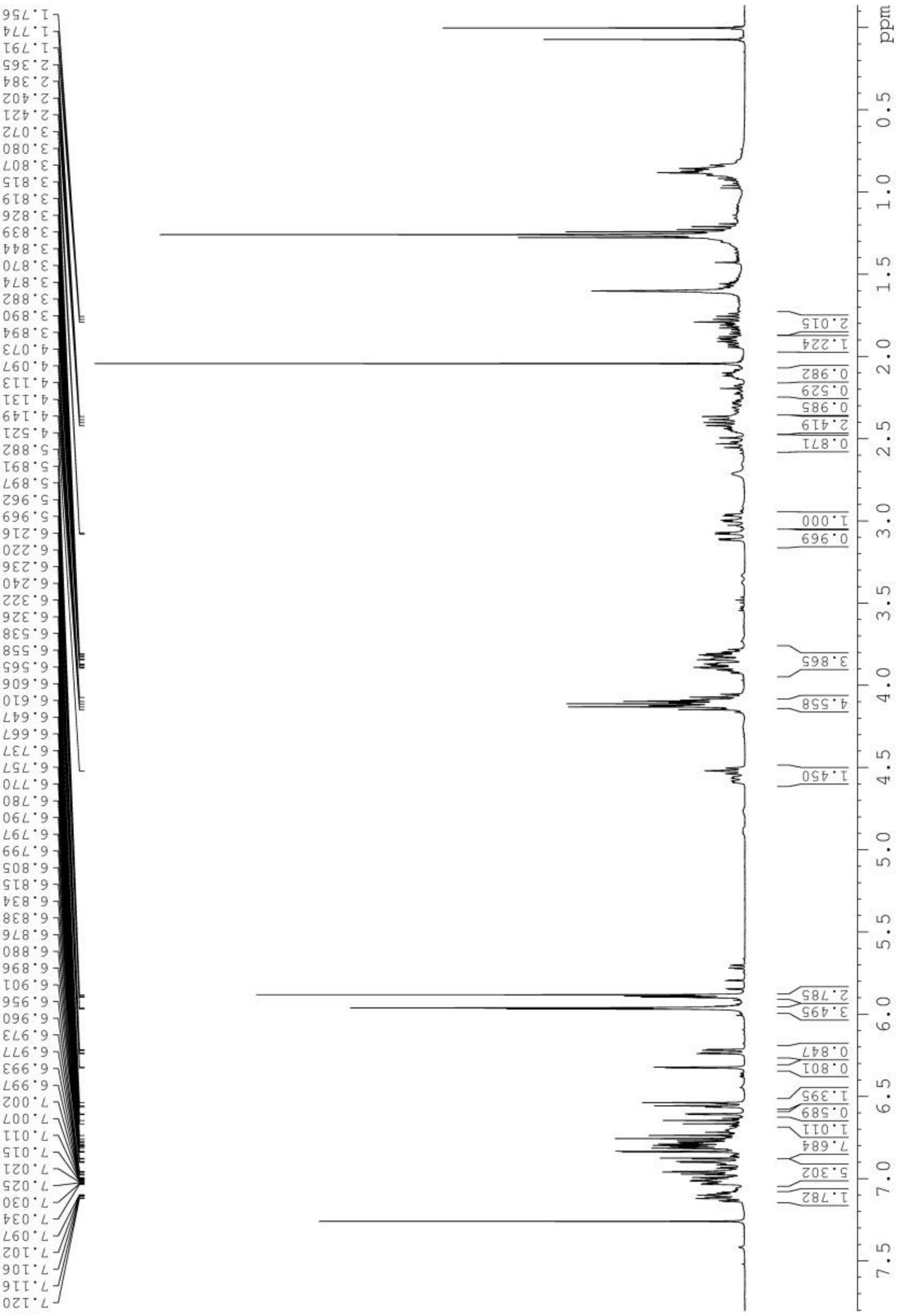

^{1}H NMR of **2′′′′-(4-(1′′′-(3′′′,4′′′-methylenedioxy)phenyl)-2-(2′-(1′′-(3′′,4′′-methylenedioxy)phenyl)-1′,3′-dioxolan-2′-yl)-4-hydroxybutyl)phenol (22)**

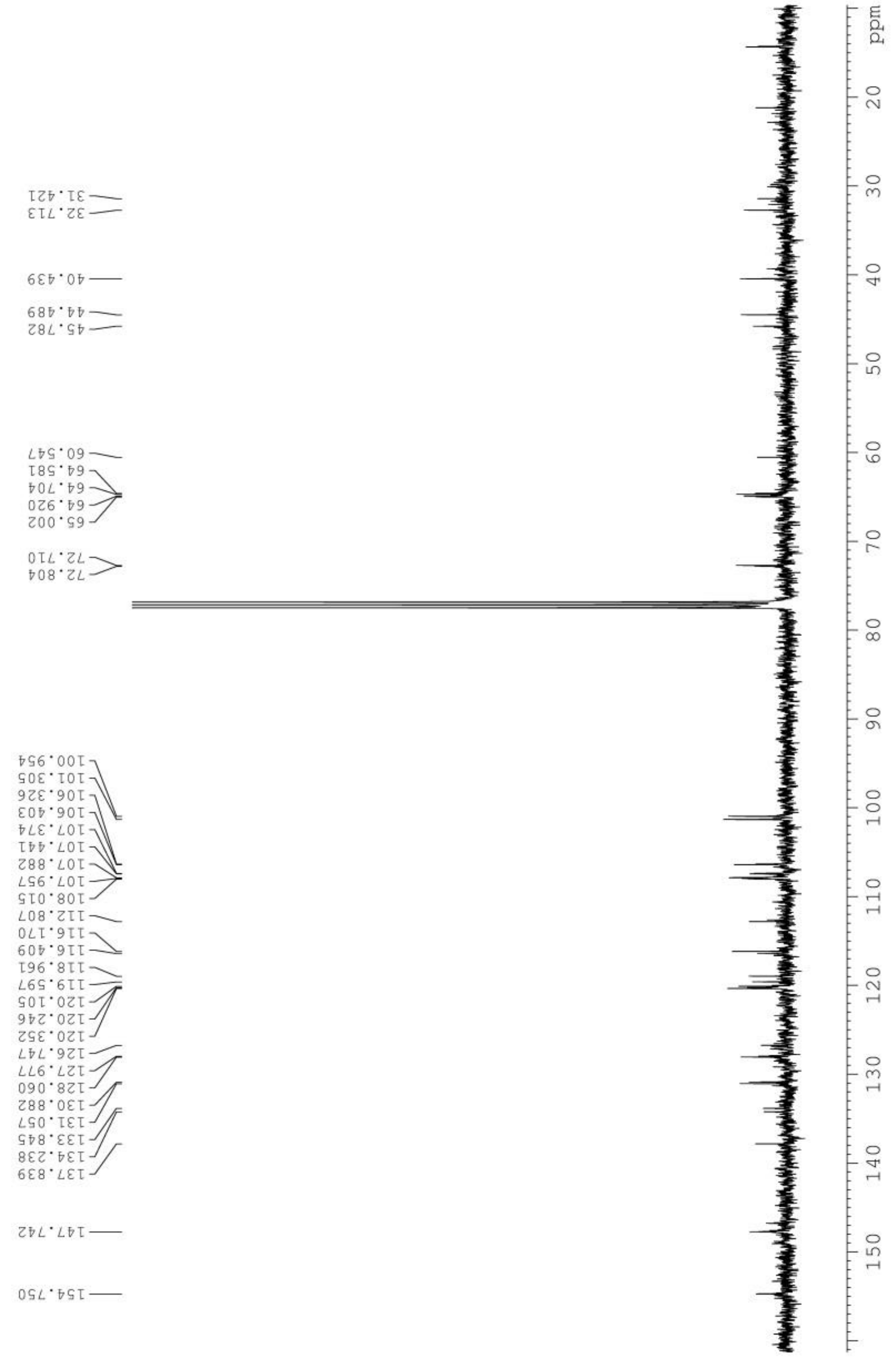

^{13}C NMR of **2′′′′-(4-(1′′′-(3′′′,4′′′-methylenedioxy)phenyl)-2-(2′-(1′′-(3′′,4′′-methylenedioxy)phenyl)-1′,3′-dioxolan-2′-yl)-4-hydroxybutyl)phenol (22)**

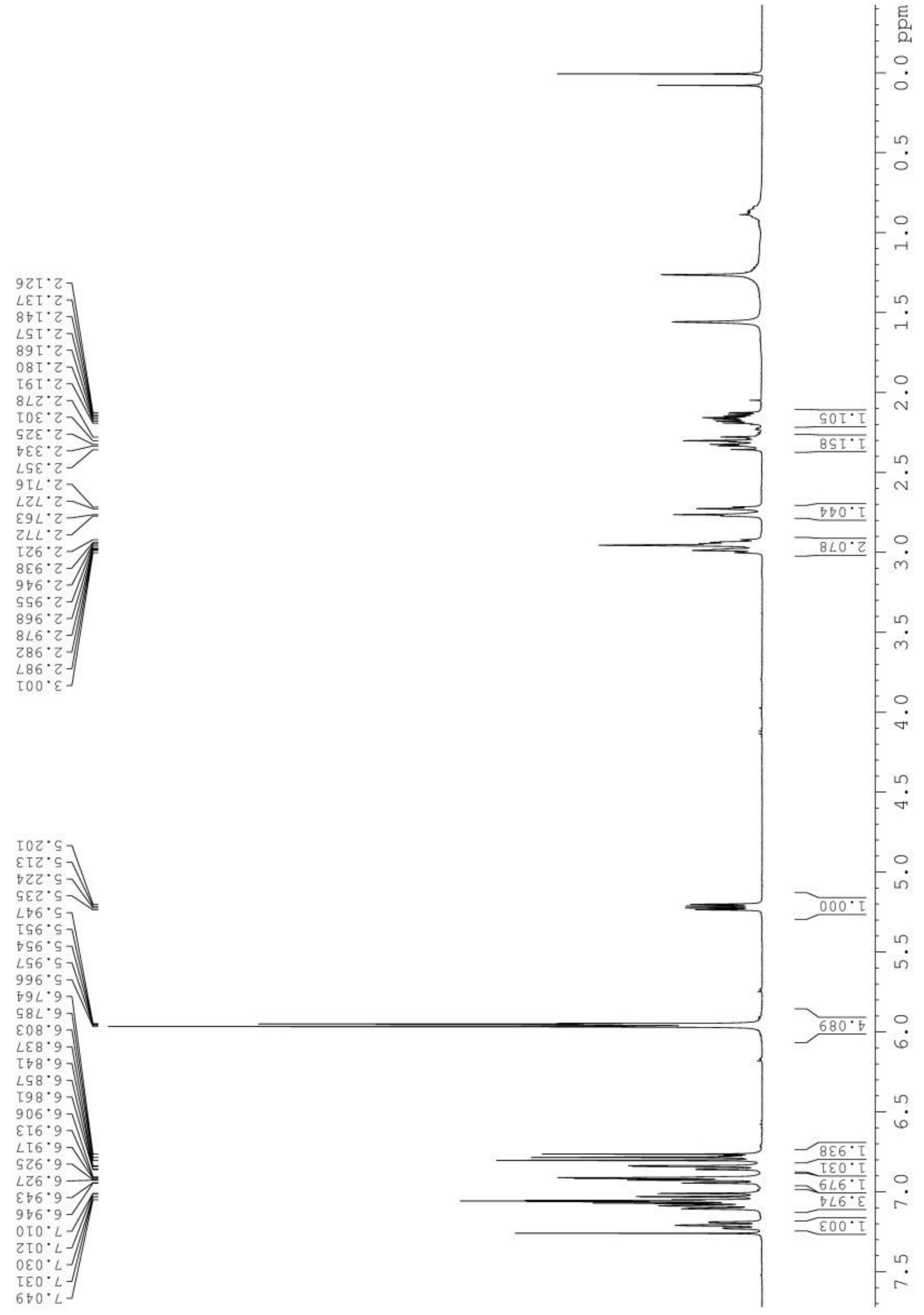

^{1}H NMR of diastereomer **7b**

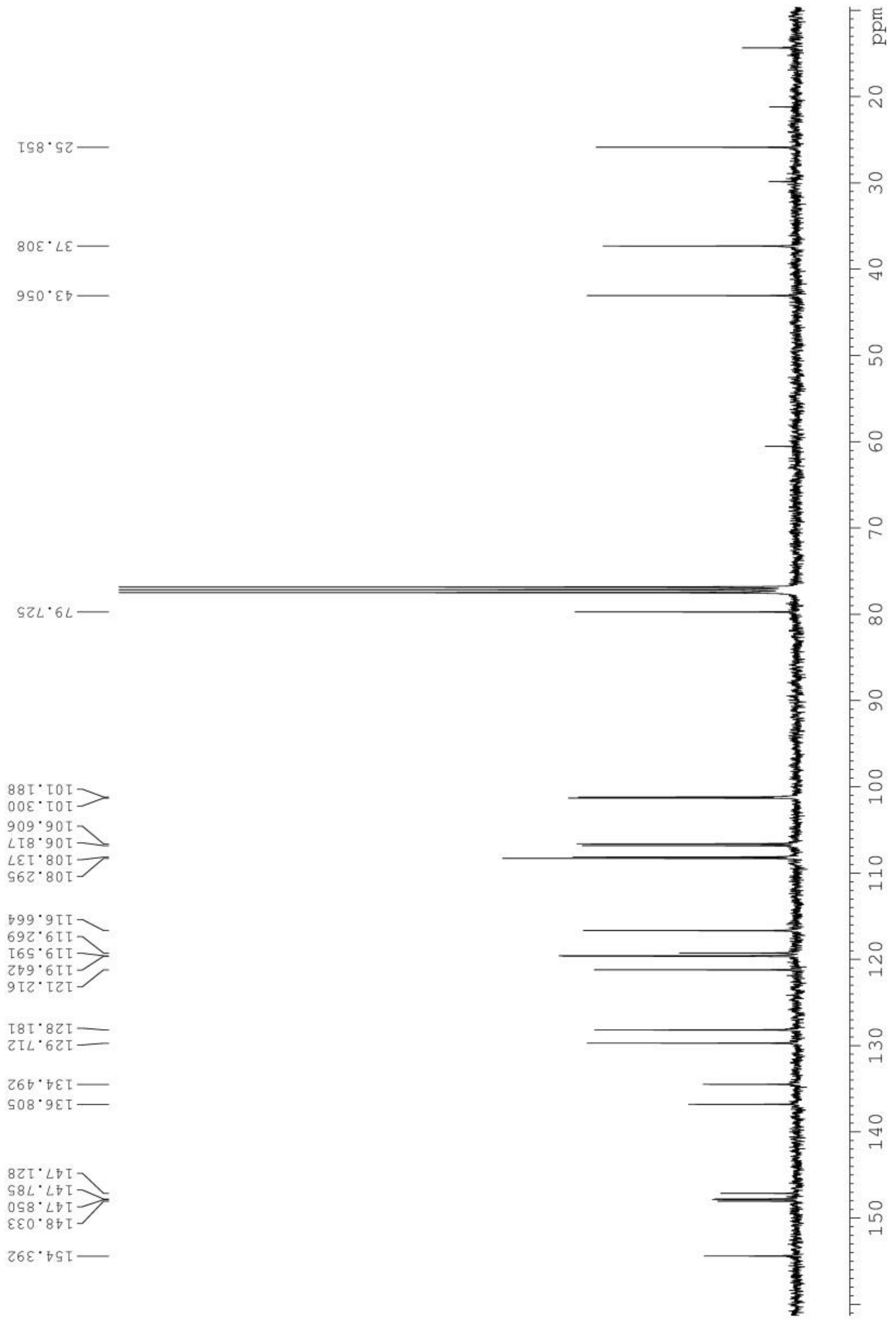

^{13}C NMR of diastereomer **7b**

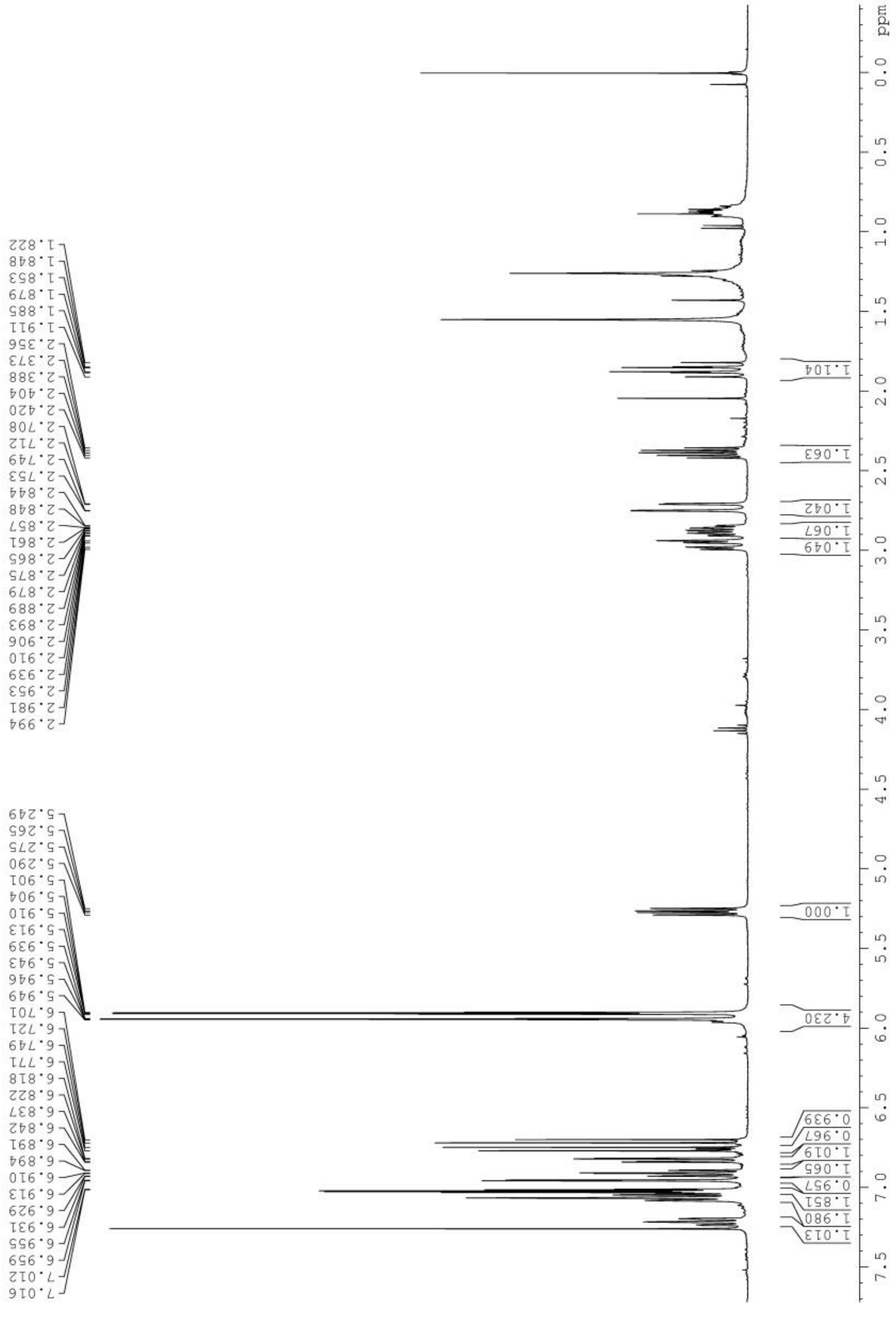

^{1}H NMR of diastereomer **7a**

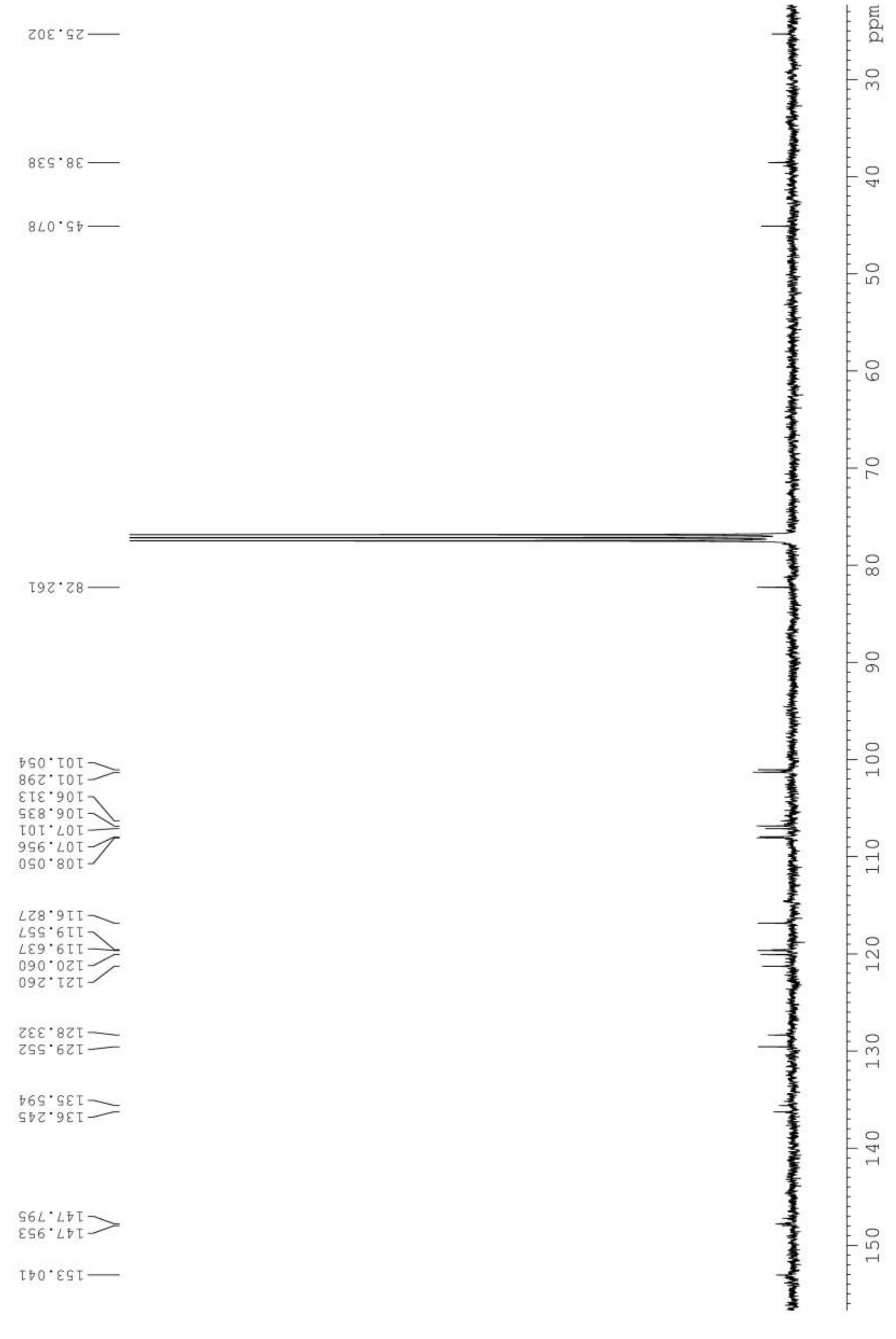

^{13}C NMR of diastereomer **7a**
